# Archaeological science meets Māori knowledge to model pre-Columbian sweet potato (*Ipomoea batatas*) dispersal to Polynesia’s southernmost habitable margins

**DOI:** 10.1371/journal.pone.0247643

**Published:** 2021-04-14

**Authors:** Ian G. Barber, Thomas F. G. Higham

**Affiliations:** 1 Archaeology Programme, Division of Humanities, School of Social Sciences, University of Otago, Dunedin, New Zealand; 2 Oxford Radiocarbon Accelerator Unit, Research Laboratory for Archaeology and the History of Art, School of Archaeology, University of Oxford, Oxford, United Kingdom; Universita degli Studi di Milano, ITALY

## Abstract

Most scholars of the subject consider that a pre-Columbian transpacific transfer accounts for the historical role of American sweet potato *Ipomoea batatas* as the *kūmara* staple of Indigenous New Zealand/Aotearoa Māori in cooler southwestern Polynesia. Archaeologists have recorded evidence of ancient Polynesian *I*. *batatas* cultivation from warmer parts of generally temperate-climate Aotearoa, while assuming that the archipelago’s traditional Murihiku region in southern South Island/Te Waipounamu was too cold to grow and store live Polynesian crops, including relatively hardy *kūmara*. However, archaeological pits in the form of seasonal Māori *kūmara* stores (*rua kūmara*) have been discovered unexpectedly at Pūrākaunui on eastern Murihuku’s Otago coast, over 200 km south of the current Polynesian limit of record for premodern *I*. *batatas* production. Secure pit deposits that incorporate starch granules with *I*. *batatas* characteristics are radiocarbon-dated within the decadal range 1430–1460 CE at 95% probability in a Bayesian age model, about 150 years after Polynesians first settled Te Waipounamu. These archaeological data become relevant to a body of Māori oral history accounts and traditional knowledge (*mātauranga*) concerning southern *kūmara*, incorporating names, memories, landscape features and seemingly enigmatic references to an ancient Murihiku crop presence. Selected components of this lore are interpreted through comparative exegesis for correlation with archaeological science results in testable models of change. In a transfer and adaptation model, crop stores if not seasonal production technologies also were introduced from a warmer, agricultural Aotearoa region into dune microclimates of 15th-century coastal Otago to mitigate megafaunal loss, and perhaps to support Polynesia’s southernmost residential chiefdom in its earliest phase. A crop loss model proposes that cooler seasonal temperatures of the post-1450 Little Ice Age and (or) political change constrained *kūmara* supply and storage options in Murihiku. The loss model allows for the disappearance of *kūmara* largely, but not entirely, as a traditional Otago crop presence in Māori social memory.

## Introduction

**Fig 1:**
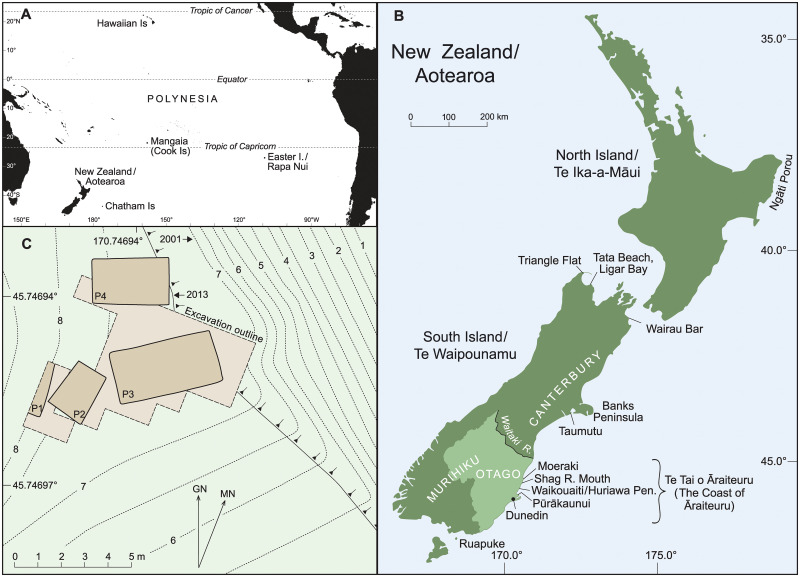
Location maps and archaeological site plan. (A) Polynesia in the setting of eastern Oceania, after map modified from SVG file ‘World map blank without borders’, GNU Free Documentation License, CC BY-SA 4.0, adding island locations from public domain map data available from U.S. Geological Survey (USGS), National Geospatial Program. (B) New Zealand/Aotearoa after map data sourced from Land Information New Zealand (LINZ) Data Service licensed for reuse under CC BY 4.0 and locations in [28,30,33,36,66,74]. Traditional Murihiku incorporates Otago region to Waitaki River (cadastral border enclosing lighter fill), as well as the southern, modern Southland region (Fig 6.8 in [30]), [33]. Shown generally only are Canterbury region north of Waitaki River and the Ngāti Porou tribal area of eastern Te Ika-a-Māui. (C) Plan of Pūrākaunui archaeological pit complex, I44/21 S dune terrace. Elevation above mean sea level (DVD1958) is determined by DGPS.

By about 1300 CE, the first human settlers had transferred ancestral Asia-Pacific crops across eastern Oceania, accompanied by the proto-Polynesian (PPN) *qariki leadership institution that gave rise to the Polynesian chiefdoms (Fig 1) [1,2]. The last took different forms, but were broadly reliant on an introduced agricultural base that integrated tree and herb crop production. In general, the dominant Polynesian agricultural modes were wet and dry field cultivation around *taro* (Araceae: *Colocasia esculenta* (L.) Schott) and *uwhi* (Pacific yam, Dioscoreaceae: *Dioscorea* spp.) geophytes respectively [3].

Polynesia’s traditional crop inventory was supplemented before 1700 CE by two American cultigens: herbaceous sweet potato (Convolvulaceae: *Ipomoea batatas* [L.] Lam.) and gourd (Cucurbitaceae: *Lagenaria siceraria* (Molina) Standl.). It seems that both crops were introduced through unknown, late pre-Columbian transpacific cultural contact(s) [2,4–7], as indicated by a few credible pre-1492 radiocarbon ages published for Polynesian *I*. *batatas* [8–10]. Thereafter, hardy *I*. *batatas* became important as a dry field geophyte in the Indigenous political economies of the cooler Polynesian margins, including the northern, ‘archaic states’ of the climatically diverse Hawaiian Islands, and the southern chiefdoms of subtropical Easter Island/Rapa Nui, marginally subtropical Mangaia (southern Cook Is), and temperate-climate New Zealand/Aotearoa (Fig 1) [2–4,8,10,11].

Among southern Polynesian islands, the archipelago of Aotearoa below S30° Latitude is novel for its largely temperate climate across a warmer to cooler north-south cline (Fig 1). In the warmest island localities of (and off) North Island/Te Ika-a-Māui, Indigenous Aotearoa Māori cultivated at least four, originally tropical Asia-Pacific cultigens, including geophytes *C*. *esculenta* and *Dioscorea alata* and two tree crops, before Europeans introduced new crops from 1769 CE [6,12–15]. Earlier 19th-century Māori grew *C*. *esculenta* in several northern South Island/Te Waipounamu locations also (p. 105–06 in [12], p. 237 in [14], p. 497, 498, 532 in [16]). More generally though, pre-1769 Māori cultivation was characterized by the production of American *I*. *batatas* (*kūmara*, used hereafter where a cultural context is specified, as are other Māori plant names) and *L*. *siceraria* (*hue*) that matured within six months, compared to *C*. *esculenta* and *D*. *alata* maturity in eight to nine months at least. The American crops were a better fit to seasonal Aotearoa where cold winters generally precluded cultigen growth, especially in southern locations [2,6,12,14]. Both *kūmara* and *hue* were cultivated in cooler Te Waipounamu also, as far south as Taumutu in eastern Canterbury at least for *I*. *batatas* (Fig 1) (p. 185 in [13], p. 38, 303 in [16]). Moreover, tuberous *I*. *batatas* roots tolerated dry soils of relatively low fertility, including coastal sands [6,12,14,15], extending production options. Harvested live *kūmara* roots were cured for long-term storage in covered semi-subterranean stores known generically as *rua kūmara* that had been invented in, and for, seasonal Aotearoa. During the later (post-1500) Māori archaeological sequence, *rua kūmara* were associated with earthworks *pā* (forts) in many places [12,14,17–21] (p. 226 in [16]).

Control of *rua kūmara* produce became an important aspect of ancient social differentiation in Aotearoa [18]. However, scholars have assumed that traditional Murihiku of southern Te Waipounamu, representing Polynesia’s southernmost permanently inhabited region, was too cold for bulk live root storage, let alone cultivation [12–14,17]. And yet, there are occasional references to *kūmara* (Te Waipounamu. *kūmera*) in the oral histories and traditional Māori knowledge base (*mātauranga*) of southern Te Waipounamu, in spite of the paucity of archaeological *I*. *batatas* evidence there (extending to a few questionable store pit records only [17]). The principal traditions refer to southern crop loss or failure, but some assume also that *kūmara atua* (deities), stores and cultivations possibly were established in the coastal Otago region of ancient Murihiku [16,22–33] (S1 Text).

An unexpected, early 21st-century discovery of pit structures in *rua kūmara* form at Pūrākaunui, coastal Otago, raises the prospect of a scientific archaeology investigation of Māori *kūmara* lore and opportunities in Murihiku. Cross-disciplinary archaeological research involving crops is not unprecedented in Aotearoa. In a “cross-field anthropology” approach, scientific archaeology, ethnobotany and historical anthropology research data have been integrated to propose that a perceived agricultural threat accounts for first contact violence between Māori and Europeans [21]. The present study extends this approach to incorporate historical science and Indigenous histories and knowledge (i.e. *mātauranga*, as above) within empirical models of internal change around the advent and loss of southern *I*. *batatas*. Data from stratigraphic, radiocarbon (^14^C) and archaeobotanical investigations and environmental science are analyzed independently to determine the age, purpose and context of *rua kūmara*-like pit structures at Pūrākaunui. Māori traditions of southern Te Waipounamu *kūmara* presence and loss and other relevant *mātauranga* are assessed and coded also from critical exegesis. These datasets are correlated thematically in testable, chronological models of change. In part, the last seek anthropological explanations of the enigmatic *kūmara* Māori lore from the south of Polynesia’s currently accepted traditional agricultural limits.

Two primary aims are achieved.

1. A credible Murihiku *kūmara* datum identifies the southern-most record of pre-Columbian *I*. *batatas* use in Oceania.
2. Southern Māori *kūmara* lore is correlated with archaeological *I*. *batatas* evidence in a novel example of ‘western’ science meeting Indigenous knowledge within a narrative mode of analysis (e.g. p.177-78, 132–33, 247, 252–68 in [2]) [21,33–36].

## Materials, methods and background

### Archaeological site and excavation

**Fig 2:**
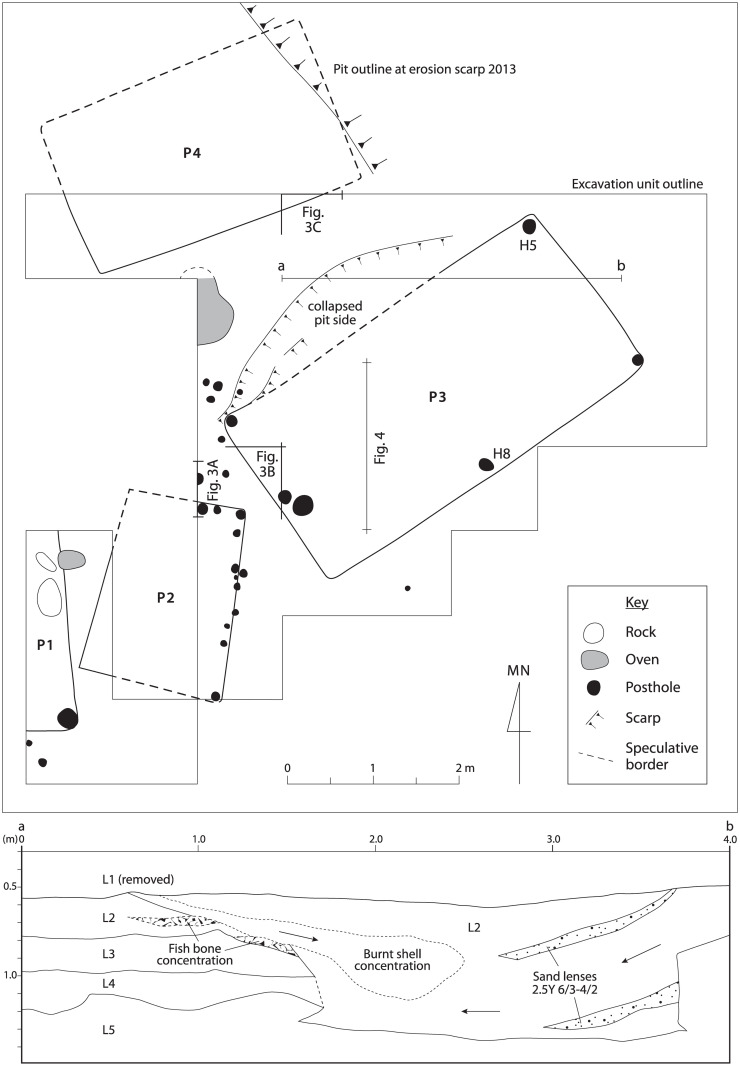
Excavation plan for I44/21 S pit structures. Pits coded P1-P4 (in bold, codes cross-referenced to Fig 1) are interpreted as subsurface components of semi-subterranean stores. All postholes recorded from the greater I44/21 S excavation area are represented. In P3, postholes from units H5 and H8 incorporating discrete starch-bearing fill are coded individually. The wall collapse along northern P3 side is an archaeological event capped by L2. Coded P3 excavation sections reference Figs 3 and 4; see also Figs 1 and S1 for further context, and S1 Table for unit descriptions.

**Fig 3:**
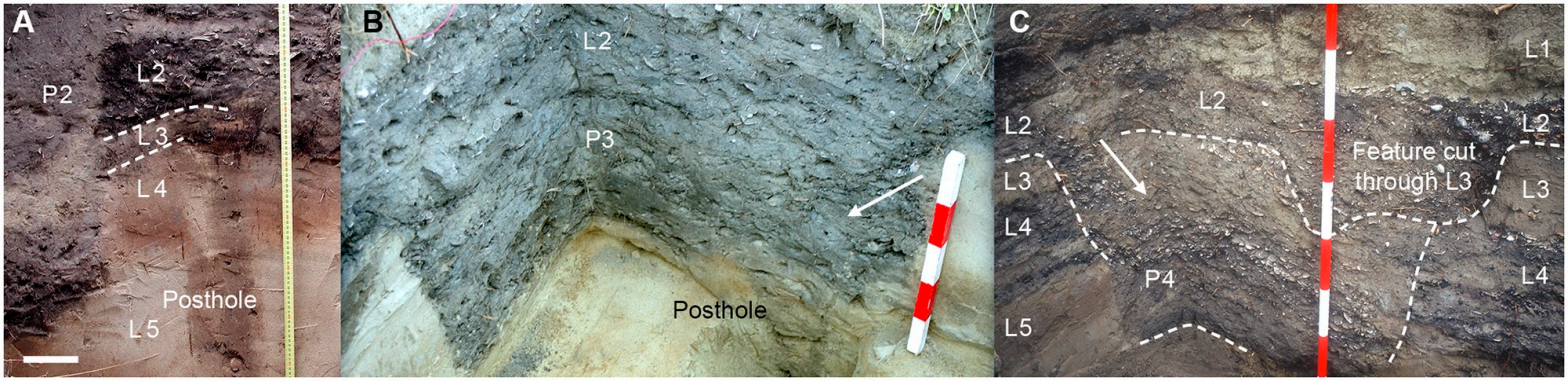
Section images from excavated pits, I44/21 S (referenced from Fig 2). Arrows indicate oblique angle of fill lenses and associated mollusc valve orientation. Unit codes follow S1 Table. (A) Northern edge of P2 truncating L2 beside external posthole (text over fill), image in perpendicular view; scale bar 10 cm. (B) Western P3 edge, unit corner and internal posthole (text mainly left of fill), oblique view; scale increment 5 cm. (C) Southeastern P4 edge, unit corner, oblique view; scale increment 20 cm.

**Fig 4:**
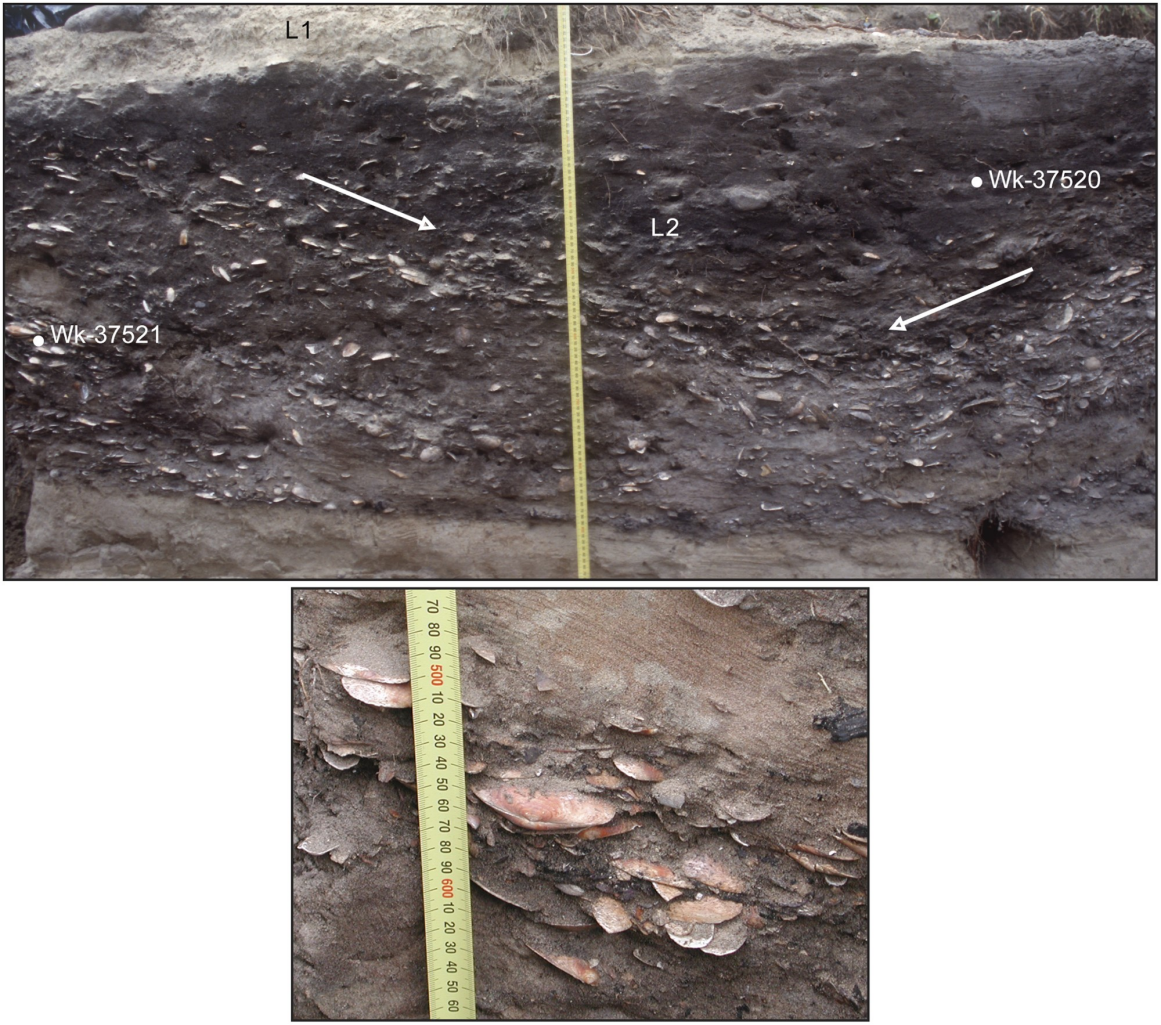
Images of P3 excavation section, I44/21 S. (Upper.) Main image, full section referenced from Fig 2, east face, perpendicular view. The locations of two dated marine samples (*Paphies australis*) are identified by ^14^C Lab numbers. Arrows indicate mollusc valve orientation in fill lenses (tape scale 10 mm increments). (Lower.) Detail of closed, articulated *P*. *australis* bivalve dated as Wk-37521 (right of tape scale at 550 mm increment), oblique view.

Māori archaeological site no. I44/21 at Pūrākaunui is a former open settlement on a sand dune system located generally more than 4 m above the channel of a tidal inlet. Site components are exposed for ~60 m along the actively eroding scarp face of the dune. These include anthropic dark sandy soils, midden sediments, animal and stone artifacts, ovens, and less frequently, pit and posthole outlines [37–41] (Figs 1–4 and S1, S1 Table).

Archaeological materials and associated data were collected from stratigraphic excavations at a designated southern area of the dune system site between 2001 and 2005 (I44/21 S). Excavation and analyses were undertaken in accordance with conditions of New Zealand Historic Places Trust Archaeological Authority No. 2001/88, complying with applicable provisions of the then regulatory *Historic Places Act* 1993. A horizontal grid of 32 contiguous 1 × 1 m square units was excavated in 100 mm intervals or spits initially (Fig 2). As excavation proceeded, spits became subordinate to stratigraphic layer units or features distinguished by discrete qualities (e.g. color, texture, matrix, borders, components; see S1 Table). Sampled soils, sediments, and biological specimens referenced by material and excavation unit have been coded digitally for curation and research access in store facilities of the Archaeology Programme, School of Social Sciences, University of Otago (UO), Dunedin. Excavation data are supplemented for I44/21 by DGPS field survey of the greater eroding site and monitoring records maintained to the present (Figs 1 and S1).

Midden analysis from this 21st-century fieldwork as well as a late 1970s excavation at I44/21 points to community reliance on local marine resources [37–39]. All midden deposits are dominated physically by marine molluscs. These are primarily bivalves *Paphies australis* Gmelin, 1790 and *Austrovenus stutchburyi* (Wood, 1828) of the soft shore channel into the inlet, with some hard shore shellfish and local, inshore finfish. Less frequently, remains of two Polynesian-introduced mammals (Māori dog/*kurī*, *Canis familiaris* and commensal Pacific rat/*kiore*, *Rattus exulans*) and native birds and sea mammals are identified. *Kurī* remains are occasionally whole but more often fragmentary, representing canine husbandry for food and artifacts. Among bird remains, modified bones of coastal or marine avifauna are most common. Elements of large, flightless *moa* ratites (Aves: Dinornithiformes, 12–249 kg) (Fig 7 in [36]) and marine seals (Pinnipedia) are generally fragmentary and scant in distribution compared to other Otago midden deposits [36–39]. Lithic artifacts include tools in eastern Otago volcanics, inland Otago silcrete and rocks sourced beyond Otago. Among the last, scattered obsidian fragments are sourced geochemically to northeastern Te Ika-a-Māui regions, as at other Otago archaeological sites with *moa* [37,40]. Carbonized plant fragments recovered from all primary archaeological layers represent a local native flora entirely, dominated by podocarps [41].

By ~1450–1500 CE, anthropic impacts across Aotearoa had driven *moa* and several large endemic rails, anatids and a Te Waipounamu eagle to extinction, and reduced breeding seal colonies in Otago (p. 75–79, 83–84 [36]). Consequently, shellfish and finfish dominance and seal and *moa* paucity from primary I44/21 deposits indicate a site chronology beyond the earliest phase of Te Waipounamu settlement, but probably no later than about the 16th century [36–39].

### Radiocarbon

Carbonized fragments of identified terrestrial plants and the carbonate fraction of marine *P*. *australis* and *A*. *stutchburyi* mollusc valves were ^14^C dated and paired for correlation and reservoir comparison. This replicates a successful dating exercise that used paired marine mollusc and atmospheric plant samples from the investigation of a ~14th century CE midden complex at Shag River mouth, about 30 km north of Pūrākaunui [42]. Samples from I44/21 S were measured using Accelerator Mass Spectrometry (AMS) or by standard Liquid Scintillation Counting (LSC) for marine samples processed before 2005.

Organic stains on marine bivalves were removed by brushing in deionized water. For earlier LSC determinations, ^14^C was measured from whole, multiple valve samples. For more recent, preferred AMS determinations, individual valves were sampled at the youngest ventral edge only, 4-5mm perpendicular to the rim, to minimize ^14^C measurement variability. Plant materials were extracted manually or by flotation from bulk unscreened samples for taxonomic and where possible, anatomical identification using voucher reference slides in UO archaeology laboratories. Intrusive roots were removed manually from specimens accepted for AMS dating. Short-life twigs or branchlets (i.e. <10 years old) identified visually by presence of pith (including cavities) and external nodes or node scars were prioritized for dating after 2013. Short-life *Typha orientalis* C. B. Presl stem materials were prepared for and imaged by a Cambridge S360 Scanning Electron Microscope (Cambridge Instruments, Cambridge, UK) fitted with Dindima Image Slave frame grabber (Dindima Group Pty Ltd, Australia) at UO (S2A Fig).

Samples selected for ^14^C dating were processed at GNS Science Rafter Laboratory and Waikato Radiocarbon Dating Laboratory, both in New Zealand. AMS pretreatments followed routine acid–base–acid procedures. All ^14^C-dated specimens and determinations are referenced by lab number (NZA for Rafter; Wk- for Waikato). Conventional ^14^C ages are reported in years BP (before present) along with δ^13^C‰ values from the source environment if measured independently by isotope-ratio mass spectrometry (IRMS).

In most archaeological research projects, including this study, conventional ages are calibrated to account for ^14^C variation by time, location and atmospheric or marine reservoirs. Calibration curves with values adjusted locally where necessary enable the conversion of conventional ^14^C determinations to calendar age ranges. We used curves SHCal20 for atmospheric southern hemisphere ^14^C and Marine20 for global marine ^14^C [43,44]. Calibrated marine dates were adjusted for oceanic ^14^C variation by use of a local, weighted mean ΔR determination (-162±28) from historic molluscs updated for Marine20 (S2 Text).

All but two of the I44/21 S determinations were analyzed further in a Bayesian age model with outlier analysis [45]. This Bayesian modeling approach has proved helpful in the resolution of relatively short, late Holocene archaeological sequences as at I44/21 (S2 Text, references). Our analysis incorporated prior information on bounded pit fill and stratigraphic units. The last were modeled as phases in OxCal v. 4.4. Each phase incorporated ages on short-life samples, including single entity mollusc valve edges, twigs or branchlets, and in one phase, a rush (*T*. *orientalis*) stem fragment. Outlier analysis was performed so as to identify and downweigh any determinations at odds with the prior framework and to exclude the posterior effect of a modeled charcoal sample with suspected inbuilt age (S2 Text). The prior was uninformative otherwise within each phase for sample type or position.

### Starch

In Oceania, attributes of reserve starch granules from storage plant parts can be diagnostic by taxon [9,46–54]. Single starch granules <100 μm long and granule aggregates were first identified in optical microscopy from secure P3 and P4 samples in 2003 (cf. precedent from a *rua kūmara* of Te Ika-a-Māui in [49]). Between 2015 and 2020, starch-bearing parts of vouched plants and archaeological sediments including further I44/21 samples were targeted for starch granule identification in a designated UO laboratory [53].

Curated archaeological sediment subsamples ~5g were prepared following standard density separation methods [55]. Mounted slides were examined and imaged by a Zeiss Axioskop 40 transmitted-light microscope with fitted digital camera. Starch granules from other archaeological sites and reference plant storage organs are published elsewhere from research associated with this project [53,54]. Additional plant reference materials were prepared in 2020. The present study targets an archaeological set of starch granules identified from P3 and P4 at I44/21 in 2016. Granule attributes were assessed against reference specimens with the interpretive control of ethnobotanical and other relevant information [9,46–54] (S3 Table).

### Oral tradition

Texts of Te Waipounamu *kūmara* traditions and other relevant Māori lore were targeted [16,22–33]. A critical exegesis was performed. The texts were assessed at source and (or) comparatively for cultural-historical integrity. Thresholds for inclusion in the model analysis of this study were replication or other verification across independent sources, and (or) association with respected authorities (S1 Text). This systematic method is broadly analogous to the descriptive-comparative evaluation of environmental science specimens by attribute fitness and context, as performed here for starch granules. Important Māori words are redefined in each separate oral tradition section for clarity.

Mythic genres were given equal weight with lineal histories and *whakapapa* (genealogies) in the selection and exegesis of Māori texts performed here. This recognizes that Indigenous narratives may weave etiological and cosmological themes into lineage histories (e.g. for Te Waipounamu and other Māori in [16,22–34] (p. 40–65 in [36]), in contrast to the secular separation and prioritization of chronologically ordered “factual” events in ‘western’ histories [35]. Moreover, Māori *mātauranga* (knowledge) may be encoded symbolically and mythically [34] (S1 Text). Accepted accounts were referenced under coded themes for comparison with historical science narratives.

## Results

### Archaeological pit discovery (I44/21 S)

Four pits with vertical sides generally and fill of midden and sand deposits were discovered on a small terrace ~6–7 m above the high tide level of the inlet channel. This site elevation precludes the deposition of *in situ* I44/21 S midden components by tidal flow or marine ingression (Figs 1 and 2). The pits and capping midden deposits were buried by dune sand >1 m deep, and not detected on the surface. Dominant molluscan and finfish components of capping, filling and basal cultural sediments are consistent with discarded food processing elements (described above). These midden sediments include articulated bivalves, finfish heads and frame sections, attesting to primary processing and undisturbed archaeological contexts. Fragmentary oven stones and angular charcoal fragments assumed to be from discarded hearth and (or) oven fill in the main are scattered through dense midden and dark sand deposits. P3 fill incorporates discrete deposits of burnt marine shell (Fig 2).

Pit 1 (P1 etc. hereafter) was excavated on the eastern side only. Smaller, almost trapezoidal P2 was excavated in discontinuous units (2.2 x 1.7 m). Larger, rectangular P3 was excavated and recorded completely (4.5 x 2.3 m) other than at the southernmost corner where unit collapse in excavation may have removed posthole evidence. Rectangular P4 (3.3 x 2 m) was excavated along its southern to western side around a unit collapse that removed the southwestern corner and (again) possible but unrecorded posthole evidence (cf. P1-P3). The northeastern corner of P4 was recorded at the eroding dune face from 2013. P3 and P4 in SW-NE orientation are filled with primary deposits from an upper midden layer (L2). P1 and P2 are oriented S-N, and P2 at least truncates L2 to post-date P3 and P4 (Fig 3). In form, size (i.e. >500 mm deep, 2–5 m long), and (for P1-P3 at least) superstructure postholes, the pits conform to the roofed, semi-subterranean, quadrangular *rua kūmara* template (Figs 2 and 3). No other principal use for Māori pits in these morphometrics is documented [12,14,17–20], while early, theoretical storage for *D*. *alata* (p. 60 in [12]) can be discounted in Murihiku where *uwhi* has never been reported or recalled (p. 228–32 in [14]) [16].

### Radiocarbon

Results are reported for all dates processed from I44/21 S (n = 24) (S2 Table). AMS marine determinations date the short-life edge of bivalves that were closed and articulated when collected manually (e.g. Fig 4). Sustained valve articulation beyond the loss of connective tissue *in situ* attests to shellfish mortality shortly before deposition and an undisturbed context since. There are no indications that marine samples have been affected by ^14^C depletion from ocean upwelling or deposit contamination, assuming local origin. Shallow Pūrākaunui Inlet is flushed by strong tides and the species dated are filter feeders, including estuarine *A*. *stutchburyi* of which North Otago archaeological samples have produced replicable ^14^C ages [42] (p. 45–48, 75 in [56]). Wherever possible, AMS marine ages have been paired by stratigraphic unit with atmospheric plant dates on short-life parts (S2 Table).

**Fig 5:**
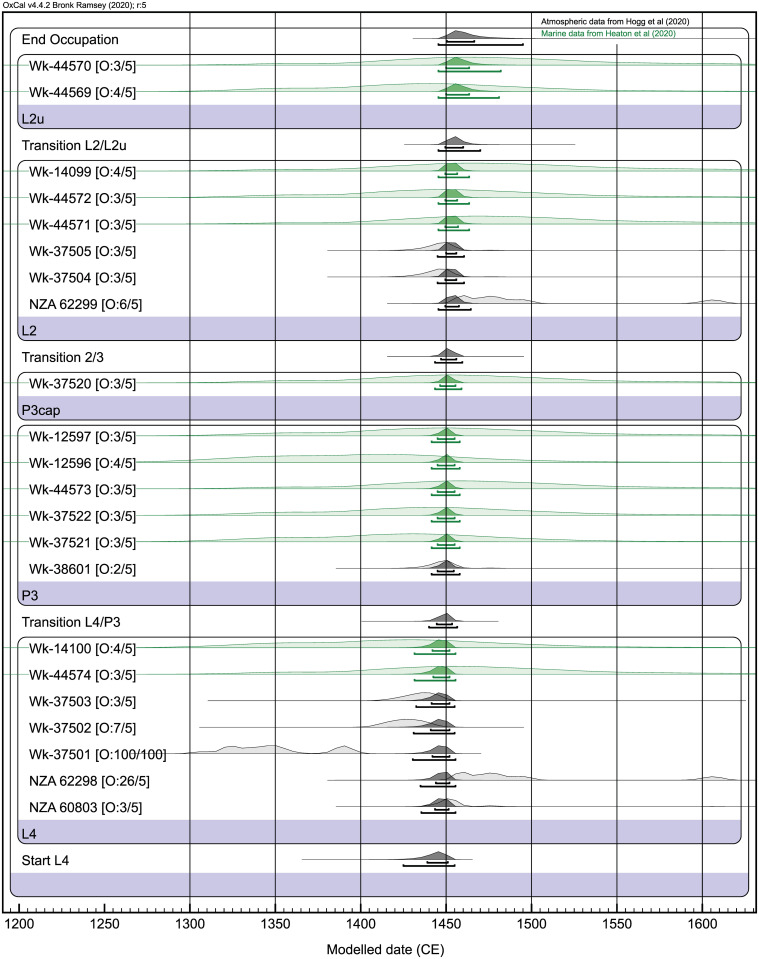
Bayesian chronological model for calibrated ^14^C ages by phase from I44/21 S incorporating Outlier [O:] analysis in OxCal v. 4.4 (after model details, code and data in S2 Text and S2 Table). The model applies calibration curves SHCal20 and Marine20 [43,44], with the latter corrected for local reservoir variation using a weighted mean ΔR value from historic mollusc data (-162±28) updated for Marine20 (S2 Text). Calibration ranges for marine (green) and atmospheric (grey) determinations present prior probability distributions in lighter fill and modeled posterior distributions in darker fill. Posterior intervals are shown below individual histograms at 68% and 95% probabilities. Prior outlier probability was set at 0.05 (5%) for all determinations except Wk-37501 (prior probability 100%), as outlined in the text.

We consider the tighter evidence of unmodeled atmospheric determinations calibrated at 95% probability first (Fig 5, S2 Table). Of four ages from the earliest cultural unit (L4) on short-life twigs or branchlets, NZA 60803, Wk-37502 and Wk-44574 fall within the 15th century CE entirely, while the greater area of NZA 62298 is distributed before 1500 (prior distributions, Fig 5). Lone, 14th-century L4 determination Wk-37501 from a carbonized and unidentified *Melicytus* sp. fragment could represent an earlier calendar event within L4. But we suspect that Wk-37501 dates an older trunk section instead, consistent with this taxon’s potential for long life and the four post-1400 CE L4 twig ages otherwise (S2 Text; cf. inbuilt plant charcoal age from I44/21 sample, p. 204 in [37]).

Secure dates from pit fill and L2 above and capping P3 and P4 are critical for *terminus ante quem* interpretations of pit construction and use. A short-life, carbonized *T*. *orientalis* stem fragment (S2A Fig) is dated from the lower fill of pit P3 in the range cal AD 1426–1477 (Wk-38601). This single entity sample may derive from rush pit lining, perhaps burnt during store disinfection (cf. 90 in [19]). Comparable intervals are reported for capping L2 twig ages cal AD 1420–1460 and 1421–1477 (Wk-37504, Wk-37505 respectively). L2 twig age NZA 62299 has a wider, multi-century distribution (cal AD 1450–1615), although the greater area of the calibrated range pre-dates 1500 CE still (prior distribution, Fig 5). Collectively, these unmodeled ^14^C distributions at 95% probability identify a 15th century CE chronology for pit construction and use.

The sequential I44/21 S chronology is tightened statistically in Bayesian age modeling with the incorporation of prior information on stratigraphy and feature relationships and outlier analysis (S2 Text references). Our four-phase model has included all standard LSC and single entity AMS determinations on *in situ* samples except for two potentially intrusive L2u charcoal ages (Fig 5, S2 Table). Of the 22 remaining Bayesian-modeled determinations, 21 were analyzed using OxCal’s General outlier detection model with a prior probability of 0.05 (5%). Age Wk-37501 only was given an outlier probability of 1.00 (100%) because of its potential inbuilt age [45] (S2 Text).

The sole outlier of any significance in the Bayesian model is NZA 62298 from L4 with a posterior probability of 26%. A single outlier in a ^14^C dataset of 21 determinations is acceptable statistically and does not affect the model outcome [45]. Posterior outlier probabilities for the 20 other ages are ≤ 7%, with 18 dates <5% probability (Fig 5), while convergence values in the greater model are >99.3. At 95% probability, the modeled start of L4 is in the range 1425–1455 CE, and the boundary transition from P3 cap to L2 falls between 1443–1459. This means that P3 was constructed and used between 1425–1459, allowing that this pit may have been excavated soon after L4 was deposited. The upper L2u boundary range is 1445–1495 (S2 Table). P2 cuts through and is later therefore than L2 (Fig 3A), but is undated otherwise, as is P1 with its unclear stratigraphic relationship to other pits. At the least, P1 and P2 attest to a subsequent, perhaps post-15th century period of pit construction following the fill of P3. The reduced size of later P2 compared to earlier P3 and P4 might represent a shift over time towards smaller storage facilities, although more examples would be needed to confirm any trend.

The I44/21 S model range of 1425–1495 CE is consistent with indications from scant and generally highly modified *moa* bone found in the same deposits that *moa* extinction was imminent if not coincident. This extinction event is identified elsewhere as occurring before ~1500 CE [36].

### Starch

As indicated above, microbotanical starch granules were identified during 2003 in sand samples of I44/21 S collected from the base of pits P3 and P4. The *in situ* basal sediments had been sampled below lenses of hard, primary molluscan midden fill (Figs 2–4). In 2016, new samples from these curated deposits were mounted and examined in light microscopy. Individual starch granules were identified again from samples of both pits, along with granule aggregates in the fill of postholes H5 and H8 from P3 (e.g. S2B Fig).

The starch granules recovered from both pits, including granules and granula masses in postholes of the P3 base >1 m apart, are interpreted as the disintegrated residue of plant parts preserved in porous basal sand from the time of storage pit use. Our study targets 34 single granules identified for assessment as possible *I*. *batatas* root starch (32 from P3 posthole H8 and two from P4 base). We have excluded the P3 posthole granule masses for assessment by taxon beyond acknowledging that they could be *I*. *batatas* also on reference information (S2B Fig).

Our assessment considers relevant reference, biogeographical and ethnobotanical criteria first. Individual starch granules from tuberous *I*. *batatas* roots in the length range 2.5–55 μm may present polygonal, circular, or semi-circular shapes (the last including forms described as cupulate or ‘bell’), pressure facets, and round or nonround and variously fissured cavities (sometimes referred to as vacuoles) at the hilum. In combination these characteristics can distinguish *I*. *batatas* starch from other Polynesian geophytes [9,46–54] (S3 Table), while Indigenous New Zealand *Ipomoea cairica* with comparable starch attributes to *I*. *batatas* is not native to Te Waipounamu (p. 153 in [47]). Discrimination may be less certain for the gourd “rind” of *L*. *siceraria* that presents circular starch granules in the length range 8.5–25 μm and a hilum with “round cavity” (p. 69 in [50] cf. [9,46–54]). However, at I44/21 pit storage is unlikely for dried gourd (*hue*) containers that were elevated above ground according to a 1919 ethnological account (p. 245, 247, 248 in [14]). Moreover, seaweed vessels (*pōhā*) had “largely taken the place of the calabash” for Te Waipounamu Māori food storage in tradition (p. 245 in [14]; see also p. 76 in [57]), [16]).

Pre-1769 Otago Māori use of several native Aotearoa plants should be considered also (S3 Table). Rhizomes of *T*. *orientalis* (*raupō*) and orchid tubers were consumed traditionally throughout Aotearoa. Even so, these and edible parts of other herbs (varying regionally) were collected generally as needed from “the bush” rather than preserved (p. 121–22 in [16], p. 68 in [57], p. 36 [quote], 69–70, 90–91 in [58]). Moreover, starch granules from fresh *T*. *orientalis* rhizomes and tubers of the most common Otago orchid genus (*Microtis*) lack a cavity at the hilum [53] (S3 Table). Dried rhizomes of the fern *Pteridium esculentum* (G. Forst.) Cockayne (*aruhe*), and stems, tap roots and shoots or rhizomes (*kāuru*) of the tree *Cordyline australis* (Forst.f.) Endl. (tī kōuka) were stored by Māori across Te Waipounamu (p. 123–25, 229 in [16], p. 120, 131–36, 144–45 in [30], p. 67–68 in [57], p. 83–84, 87–89 in [58]) [59,60]. *Kāuru* is not relevant since its storage carbohydrate fraction is fructan [61], and diagnostic *Cordyline* spp. starch granules are not reported otherwise (p. 154 in [47]). Te Ika-a-Māui reports do describe *aruhe* kept in *rua* or “store pits” on occasion (p. 81 in [58]), and dried, starchy rhizomes of the bindweed *pōhue* (*Calystegia sepium* subsp. roseata) “stowed in a storehouse” in baskets (p. 90 in [58]). However, *P*. *esculentum* starch granules are distinctively oval or “teardrop” in shape and do not have a cavity at the hilum (Fig 3 in [45], Fig 10 in [53]). Starch granules of *C*. *sepium* rhizomes (*pōhue*) can overlap *I*. *batatas* root granules in shape and size, but the former lack cavities at hila also (p. 205, Fig 10 in [53]). As well, *pōhue* is not a Māori food recognized in traditional records published for Murihiku (p. 117–25, 194 in [16], 133, 134–35 in [30]).

Starch granules of one other native Aotearoa plant should be considered. Early Polynesian colonists relocated Te Ika-a-Māui tree *Corynocarpus laevigatus* J.R.Forst. et G.Forst. (*karaka*) as far south as Banks Peninsula for its nutritious starchy seeds that could be “stored away in baskets” for years once processed and dried (*kōpīa*; p. 38, 192, 302 [quote] in [16], p. 120 in [30], p. 46 in [58]) [53,59]. The early distribution of *kōpīa* to Murihiku is conceivable (if undocumented), as is consequent archaeobotanical confusion with *I*. *batatas* starch in analysis. Circular starch granules of *I*. *batatas* and *C*. *laevigatus* both present hila with cavities that are sometimes fissured. Of these, *I*. *batatas* would be distinguishable only above the maximum reported *C*. *laevigatus* granule length of 13.2 μm where applicable (p. 203 in [53]; S3 Table).

**Fig 6:**
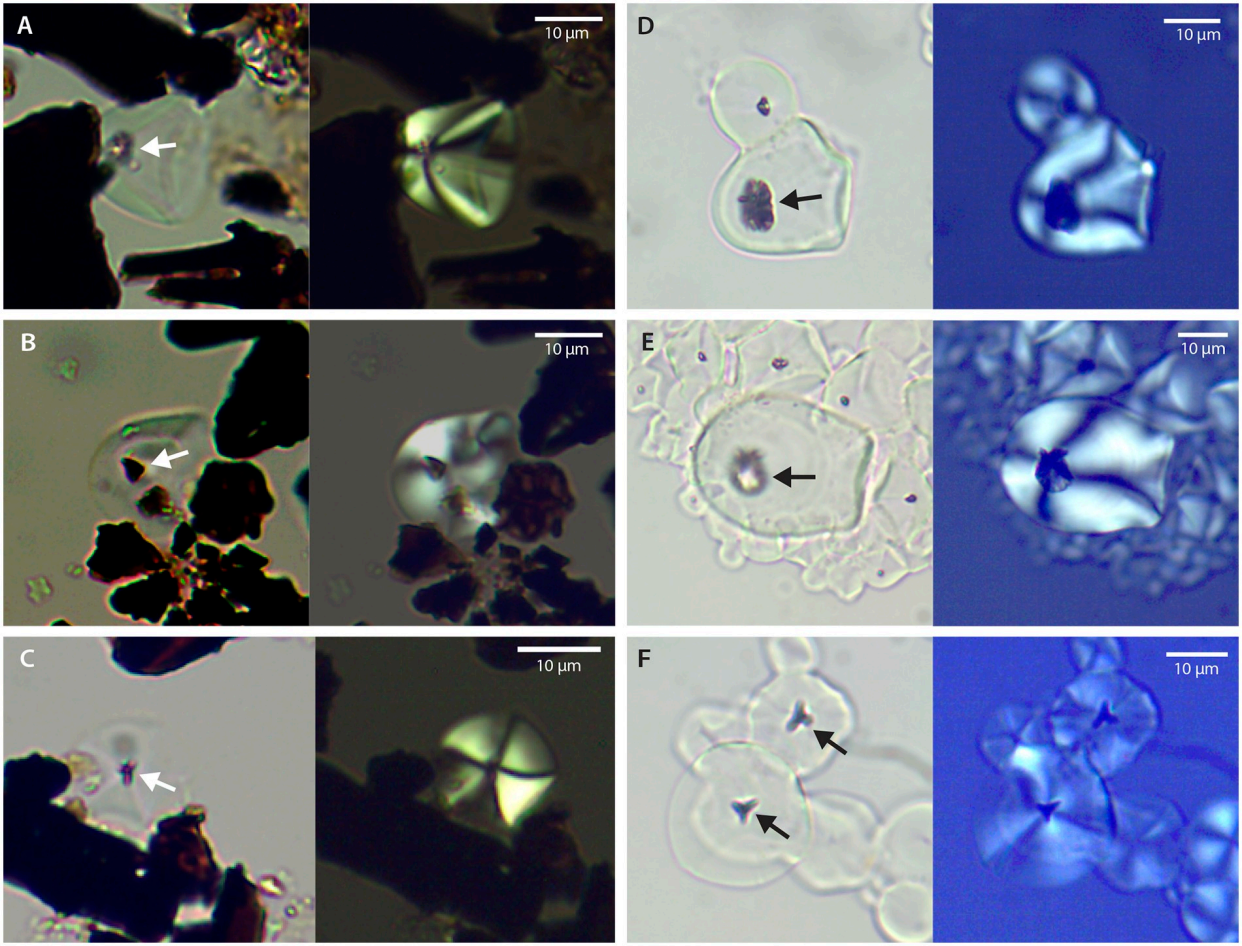
Starch granules from P3, unit H8 posthole fill (I44/21 S, panels A-C), and from reference *I*. *batata*s roots (D-F). Paired images in brightfield (left) and polarized light (right), with the hilum shown at the intersection of a birefringent extinction cross in polarization. Arrows point to nonround cavities and fissures at the hila of granules >13.2 μm. In granules of two panels (C, F), hila of archaeological and reference specimens present with distinctive y-shaped fissures reported previously for reference *I*. *batatas* (Fig 2a in [46], Fig 9e and 9f in [51]).

Informed by this review, we focus on 31 circular to semicircular or polygonal granules >9.0 μm in length identified from I44/21 S contexts in 2016. These are unlikely to be from preserved *taro* corms on size or from *P*. *esculentum* on shape, while 15 specimens >13.2 μm preclude assignment to *kōpīa* (S3 Fig, S3 Table). Attribute comparison strengthens the likelihood that some if not all of the largest granules in this subset >13.2 μm from a basal *rua kūmara* context are *I*. *batatas*. Here we compare a North American study of *Solanum jamesii* starch granules from archaeological stone tool edges that applied a descriptive-comparative method similar to our own. These researchers identified 61 granules as “likely or possibly” *S*. *jamesii*. Nine further granules only were assigned to *S*. *jamesii* on reference criteria (p. 7606, S3 Table in [62]). From I44/21, granules of a set numbered similarly to the last (n = 8; P4 base x 1, P3 H8 posthole fill x 7) are identified as likely if not probable *I*. *batatas* with combined specimen attributes of shape (as above), size >13.2 μm, and distinctive cavities at the hila that are often fissured (Table 1). Cavities or vacuoles and fissuring at the hila are not described in any of the larger granules of native, edible starchy geophytes of record as noted above (see also S3 Table), but are considered highly characteristic of *I*. *batatas* (e.g. Fig 6, Figs 2a and 4a in [46], Fig 9e-9g in [51], Fig 7e in [52]).

**Table 1:** Length and cavity attributes of starch granules >13.2 μm from I44/21 S pit contexts.

Sample context (Pit no./unit code)	N	Maximum length range	Cavity/fissure at hilum^a^
Basal sediment (P4/K5)	1	15.7 μm	1
Posthole fill (P3/H8)	14	13.6–24.2 μm^b^	7

^a^ Eight granules with cavities/fissures at hila represent 53% of total I44/21 S granules >13.2 μm (n = 15) identified in 2016 set. Three granules from P3 present nonround cavities or fissuring at the hilum (Fig 6).

^b^ Size range is inclusive: See S3 Fig for distribution of all measured P3 granules.

Potential *I*. *batatas* assignment in the 2016 set is most compelling for three granules from H8 (Fig 6). These present strong *I*. *batatas* reference similarities in overall form and nonround hila each, including a distinctive, fissured, “y-shaped” cavity published previously (Fig 6C and 6F cf. Fig 2a in [46], Fig 9e and 9f in [51]).

### Oral tradition

*Kūmara*/*kūmera* references are embedded in foundational oral traditions published from the principal Te Waipounamu Māori tribal collective Ngāi Tahu (S1 Text). These traditions and associated *mātauranga* (knowledge) appear to include narratives both transferred directly from Te Ika-a-Māui and incorporated from earlier Te Waipounamu *iwi* (tribes) Ngāti Māmoe (southern var. Kāti Māmoe) and Waitaha [30] (p. 135–82 in [32]). Published traditions were selected for comparative analysis in this study on the basis of critical text assessment (S1 Text).

Across this southern lore there are multiple references to the root noun Rongo (southern var. Roko), the mythic and sometime culture hero source or *atua* (influential ancestor, deity) of *kūmara*/*kūmera* in Aotearoa, and crops elsewhere in Polynesia (as Lono) [3,21], (p. 103, 111–12 in [31], p. 174–77 in [32]). Early mythic peoples of the Polynesian homeland Hawaiki if not Te Waipounamu also are identified in several independent southern (including Murihiku) accounts by Roko or Rongo names, including the group name Kāhui Roko (Kāhui = swarm, cluster, company) (S1 Text). In one account, Kāhui Rongo introduce *kūmara* to Aotearoa from the Polynesian homeland Hawaiki (v.31 p. 195 in [27]; see also p. 176–77 in [32]). These expressions of Rongo localization represent the successful introduction and maintenance of *kūmara mātauranga* in Te Waipounamu, even in southern localities where *I*. *batatas* production would have been impossible (e.g. Ruapuke island in [31]).

One southern Rongo/Roko narrative stream highlights the values of, but also, the challenges in securing southern *kūmara*. Multiple Ngāti Māmoe *whakapapa* (genealogies) identify Roko i Tua who is associated with a widespread Māori narrative of *kūmara* origins as an ancestor (v.24 p. 33 in [27], p. 15, 23 in [30], p. 175–76 in [32]). In the core myth, Rongo from Hawaiki visits Kāhui or Toi people in Aotearoa who offer him wild plant foods (e.g. p. 160–62 in [23], p. 61–62 in [29], p. 15, 22 in [30], p. 102–20 in [31], p. 174–77 in [32]). Rongo declines the local foods. Instead, he rehydrates dried *kūmara* (*kao*) from Hawaiki that he offers to the Aotearoa people. The taste of *kao* so impressed the last that they (or Rongo) provision variously identified *waka* (canoes) named Manuka, Ārai Te Uru (in southern accounts only) or Horouta (primarily in Te Ika-a-Māui versions) to source live Hawaiki *kūmara* (S1 Text).

Although these *waka* all return to Aotearoa with *kūmara*, crop establishment was unimpressive if not unsuccessful in Te Waipounamu accounts. *Kūmara* from Manuka failed to produce (p. 161 in [23], p.12 in [24 v.3], p. 18 in [25], v.24 p. 108 in [27]), rotting “in the ground” in a Murihiku version because the required *karakia* (ritual incantation) was neglected (v.24 p. 108 in [27]). From a Canterbury authority, Horouta and Manuka returned with a “straggly few” *kūmara* only, leaving later *waka* to bring the “better kinds” (p. 62 in [29]). In a widespread Ngāi Tahu account, Ārai Te Uru returned to lose its *kūmara* cargo overboard finally in stormy seas off Moeraki, North Otago, before the *waka* wrecked on the coast of its traditional name (S1 Text). The lost *kūmara* eventually became “globular” North Otago beach boulders as identified by local Māori in 1844 (p. 15 in [30]; see also S1 Text).

These Ngāi Tahu accounts of various crop disappointments or mishaps from Manuka, Horouta and Ārai Te Uru *waka* are coded ‘*kūmara* loss’ collectively (Table 2). This code designation is strengthened comparatively against Te Ika-a-Māui accounts of the core tradition. In the last, *kūmara* distributed at several locations in Aotearoa from the repaired, returning Horouta *waka* produced successfully (p. 102–03 in [24 v.3]), p. 137–46 in [28], p. 40 in [63]), [64] (S1 Text). One Te Ika-a-Māui account even recalls “the abundance” of *kūmara* from the place where Horouta was finally emptied in a traditional saying: “Behold the greatness of the output of the hold of Horouta” (p. 144 in [28]).

**Table 2:** Murihiku Māori and archaeological *kūmara* themes and chronologies correlated horizontally, after article model and S1 Text (? = interpretation tentative, all chronologies CE).

**Code**: Māori tradition, ethnography	Scientific chronologies	Model theses by integrated chronologies
***Kūmara* transfer, success**:	~1400–1500 (from ^14^C-dated archaeol.): megafaunal loss	1430–1460 ^b^: *rua kūmara* introduced at warm coastal locations—possible mitigation for faunal loss
Mythic Kāhui Roko^a^ in multiple southern accounts (including Murihiku), introducing *kūmara* in some	1430–1460 ^b^: I44/21 *rua kūmara* structures with *I*. *batatas*-like starch	
Ngāti Māmoe *iwi* (tribe) to Te Waipounamu from/after ~1500, Roko i Tua as ancestor	In (or from?) 15th century, as above: I44/21 *rua kūmara* in stratigraphic sequence	1430–~1650(?)^c^: coastal *kūmara* stores (& production?) sustained: LIA impacts potentially from ~1450
***Kūmara* loss**:	~1450–1850 (environmental dating): New Zealand LIA, absence of credible surface *rua kūmara* in Otago archaeol. record	~1650(?)-1800^c^: live plant foods generally native; sustained LIA effects & (or) politics disrupt availability of live, storable *kūmara*
Otago *kūmara* stored (Te Wera, ~18th century), *atua kūmara* (deities) kept at Waikouaiti only		
Ngāi Tahu Rongo i Tua/*waka* traditions amended		

^a^. “Esteemed tribe” of Rongo/*kūmara*, after p. 176 in [32].

^b^. Bayesian age model for I44/21 S.

^c^. Traditional generations for ~1650; documentary/postcontact history for 1800, as per text references.

However, independent traditions tell a different story of southern *kūmara* from a *pā* (fortified place) at “Waikouaiti”, North Otago (assuming Huriawa Peninsula, Waikouaiti river mouth, less than 20km north of Pūrākaunui; see S1 Text). In a 19th century Ngāi Tahu account, *kūmara atua* (deity) Rongo/Roko Nui a Tau who was kept at Waikouaiti *pā* was stolen during a siege (p. 185–87 in [24 v.3]). To honor his missing *atua*, the “priest” of the *pā* cleared a small space “to resemble a cultivation” on which mounds were made like those for a ritual *kūmara* garden (e.g. p. 116–19 in [14]). This mimetic ceremony facilitated Rongo’s supernatural return (p. 186–87 in [24 v.3]). At the same location apparently an independent 1906 history drawing on local Māori sources identified “cultivations” below Te Pā a Te Wera (the *pā* of Te Wera) (S1 Text) [26]. This last memory is associated with a later 18th century siege of the *pā* by Te Wera’s relative Taoka, during which the rainbow deity Kahukura was stolen. However, Kahukura returned miraculously in response to *karakia* (ritual incantations) [26], like Roko Nui a Tau. Kahukura is associated traditionally with Rongo Nui a Tau in fact, and is considered a source and *atua* of *kūmara* also (S1 Text). In Te Ika-a-Māui versions of the southern *kūmara* origins tradition, Kahukura even partners with if not replaces Rongo i Tua, especially among east coast Ngāti Porou who are cognate with Ngāi Tahu (Fig 1) (p. 157 in [14], p. 103–04 in [24 v.3], p. 68–69, 132–46 in [28], p. 21–23 in [33], p. 40 in [63], p. 415 in [65]) [32,64]. Of particular note, *kūmara* is listed among Pā a Te Wera foods stored against the threat of siege in the well-regarded Ngāi Tahu history complied by Carrington (p. 138 in [33], S1 Text). This account implies that *kūmara* roots were available locally (along with the other listed resources) so that Te Wera’s *pā* could be “well stocked”.

The association of two ancient *kūmara atua* at this Waikouaiti location linked to Te Ika-a-Māui ancestors supports the antiquity and successful transfer of *kūmara* knowledge if not the crop itself to Murihiku. The last is suggested independently in the Carrington account of 18th century *kūmara* stored at Pā a Te Wera. But comparatively, the regional 18th century novelty of *kūmara* storage at Waikouaiti contributes to the broader theme of loss as well (Table 2). It has no equivalent in post-1650 ethnohistory or ethnography of record for any other site from Otago [16,30,57].

## Discussion and narrative models

Following our Bayesian age model, I44/21 storage pit construction and use events associated with preserved *I*. *batatas*-like starch fall within the posterior decadal interval 1430–1460 CE at 95% probability, around 150 years after the first Polynesian settlements of record are dated in Te Waipounamu [36,42,56,66]. This archaeobotanical chronology from the dunes of Pūrākaunui can now bolster the small number of credible Oceanic indicators of late pre-Columbian *I*. *batatas* reported elsewhere in Polynesia [e.g. 8–10].

The location of this Pūrākaunui *rua kūmara* discovery at the unlikely latitude of S45.75°, ~250 km southwest of the confirmed archaeological record for *I*. *batatas* cultivation or live root storage (S43.83°) [17], is more remarkable even than its pre-Columbian chronology (Fig 1). But if unprecedented, the discovery is not inexplicable. Live *I*. *batatas* roots become highly susceptible to fungal rot when average daily temperatures drop below 10°C [20], as was typical of Otago’s earlier 20th-century winters [67]. However, the regular “intervention” of internal fires and heated rocks in a covered pit might maintain interior storage temperature above 10°C in these situations [20]. Large rocks and evidence of fires at the base of several archaeological *rua kūmara* in Te Waipounamu may be evidence of such active management [17,20,21]. At I44/21, oven features and stone fragments are common, and two massive basalt beach boulders were recovered from the base of P1 [37–39] (Fig 2).

The source of proposed *I*. *batatas* at I44/21 is more puzzling. In Ngāi Tahu lore, preserved (non-living) *kūmara* or *kao* were exchanged across Te Waipounamu networks in reciprocal feasting (*kaihaukai*; p. 278 in [16], p. 126–28 in [30]), and are identified, as above, in southern Roko i Tua accounts. An early 20th-century source assumes also that *kao* was distributed from Canterbury as far south as Murihiku through traditional *kaihaukai* events (p. 67 in [57]). However, *kao* can survive cold winter temperatures with no special seasonal storage requirements in Murihiku (unlike live *kūmara* roots; see S4 Fig, including caption), whereas the construction of *rua kūmara* several meters long at I44/21 points to the storage of live, cured, tuberous roots in bulk [cf. 17–21]. In the Ārai Te Uru narrative, bulk root transfer into cooler southern waters is possible but precarious. This is underscored in a 19th century description of *kūmara* prepared for storage: “a very short exposure to damp, or even to cold air, will quickly spoil the whole lot” (p. 224 in [14]). The sustained supply of live *kūmara* roots over hundreds of kilometers by sea would have been a remarkable accomplishment.

Could *I*. *batatas* have been grown in Otago instead, as Waikouaiti traditions might imply? Certainly, ongoing store construction at Pūrākaunui is consistent with cultivation propinquity. But the lands of both I44/21 at Pūrākaunui and Waikouaiti River mouth fall into generalized climate zones with median summer (January-March) temperatures in the range 14.1–14.5°C as measured 1.3 m above ground and 10 cm below ground [68]. Reliable *I*. *batatas* tuber development requires soil temperatures above 15°C at least [69,70]. Even so, the modern climate of Dunedin, <20km south of Pūrākaunui, is spatially variable with warm microclimates and mean summer earth temperatures at and above 15.5°C, allowing subtropical cultivation in some locations (p. 28, Tables 17 and 18 in [71]). Consistently, several kg of tuberous *I*. *batatas* roots were produced from an outside Dunedin home garden in 2018 [72], while tuberous root development is documented from *I*. *batatas* grown under cover in a relatively warm, north-facing Dunedin location between 2019–2020 (S4 Fig).

Ancient *kūmara* varieties may have been selected and acclimatized for cold tolerance and rapid tuber development in the root as well (e.g. [6], p. 26 in [20]), at least prior to adverse effects of the Little Ice Age (LIA) after ~1450 CE ([73] and climate discussion below). Here it is relevant to note the resemblance of a dark, mixed anthropic sandy soil with basal depressions exposed briefly during 2007 in a dune erosion scarp 40m north of P4 at I44/21 to *kūmara* “planting pit” profiles in mixed, sandy, pre-1769 cultivation soils of northern Te Waipounamu (S5 Fig) (cf. [6,21,47,74]). Regrettably this I44/21 scarp profile disappeared before a soil investigation could be undertaken. This means that 15th century microclimate *kūmara* production in the relatively warm dune sands of Pūrākaunui is an intriguing but unconfirmed archaeological possibility only at present (Table 2).

Two southern *kūmara* models that recognize these uncertainties are outlined below. Both models correlate relevant archaeological science, tradition and *mātauranga* (traditional knowledge) in testable, narrative explanations of change (cf. “literary” or “discursive” model forms in [75]), with reference to Table 2).

### Model for Otago *kūmara* transfer and adaptation

*Rua kūmara* are not identified in excavation records of the earliest, ~14th century Otago settlements, nor Wairau Bar even, the oldest, securely dated Te Waipounamu village (earlier 14th century) (p. 75–80 in [36] and references) [42,56,66]. Crop store absence at Wairau Bar in a region of (later) archaeological Māori and modern *kūmara* cultivation seems especially notable [70]. The initial availability of abundant megafauna may have discouraged marginal crop production and storage for these older, eastern to southern Te Waipounamu settlements. However, the earliest Polynesian settlers in other warm, especially northern Aotearoa places with arable soils and relatively sparse *moa* populations appear to have been more reliant on adapted topical agronomy, including northwestern Te Waipounamu [15,47,48,54,74,76]. Consequently, this model assumes that regionally unprecedented mid-15th-century *rua kūmara* at Pūrākaunui represent the southern transfer of a crop store technology developed previously in a warmer, agricultural Aotearoa climate.

Oral tradition offers a plausible sociopolitical correlation. Te Waipounamu *whakapapa* (genealogies) replicated across multiple *whānau* (family) records and restructured in the form of ‘western’ chronologies identify the southern migration of a succession of east coast and lower Te Ika-a-Māui *iwi*. From whakapapa records alone, this began very approximately “after about AD 1500” (p. 62 in [33]; see also [30,32]). Among the first recorded migrants, ancestors of Ngāti Māmoe with *kūmara*-carrying forebear Roko i Tua, as documented above, arrived among an *iwi* (Waitaha) with their own traditions of early Kāhui Roko in the 19th century record at least (p. 269–72 in [32], adapted perhaps to accommodate Waitaha primacy).

These correlations provide for two modeled theses (Table 2). In a mitigation thesis, the earlier 15th-century Otago importation of novel *kūmara* stores countered the loss of local high energy food from predation-induced population depression in megafauna (p. 83–84 in [36]) [66]. Sustained pit construction at I44/21 S, possibly beyond the 15th century, suggests some measure of success in the supply, if not local production, of acclimatized *kūmara* with values of storability and nutrition [69,70]. A sociopolitical thesis would allow that these *rua kūmara* may have been under elite management so as to reinforce social differentiation in the early (pre-*pā*) Murihiku political economy (p. 91–103 in [30], p. 29–32 in [33]). This last thesis assumes that *kūmara* were perceived as a culturally prestigious food of sacred Hawaiki as echoed in ancient southern traditions (p. 103–19 in [31], p. 169–77 in [32]).

Qualitative and quantitative tests of these theses are proposed.

- Stable isotope analysis to test assumptions of changing human diet and resource availability between 14th and 15th century Otago sites (anticipated in [77]).
- Surface, subsurface and remote sensing surveys of coastal Otago sites to search for and date arable soil use and *rua kūmara*, as well as relevant microbotanical remains.
- Comparative analysis of site layout, material culture and exchange involving Otago archaeological places with proposed *rua kūmara* to test for social differentiation.

### Model for Otago *kūmara* loss

This model recognizes that in spite of the cultural maintenance of ancient *kūmara* lore around the mnemonic Āraiteuru coastal landscape, Beattie’s early 20th century “southern Maori” ethnological field project does not reference *kūmara* storage or cultivation memories south of Taumutu, Canterbury (p. 38, 303 in [16]; also v.27 p. 148 in [27,57]). This is consistent with the ambiguity around the few possible records of surface *rua kūmara* in Otago archaeology [17].

The loss model tests a possible variable in which cooler temperatures incident to the global LIA (~1450–1850 CE in Aotearoa [73]) led to production failures and impacted Otago *kūmara* supply around, and from, newly vulnerable 15th to 16th century horticultural margins, wherever they may have been precisely (p. 27 in [33], p. 119–20, 123–24 in [36]). If so, 21st century *I*. *batatas* propagation in Dunedin [72] (S4 Fig) might flag the climate change return of a pre-LIA Murihiku adaptation. As well, or even alternatively, adverse LIA weather events may have impacted bulk *kūmara* root transfer to Otago by *waka* (canoe), a possibility with resonance in the tradition of the Ārai Te Uru upset.

In this climate explanation, LIA effects sustained into and beyond the 16th century account for the development of southern founding *waka* narratives of *kūmara* loss, and the general, although not complete disappearance otherwise of a live southern *kūmara* presence from social memory. Beyond possible Waikouaiti (Huriawa Peninsula) *kūmara* stores, traditional records for the period from ~1650 indicate that Murihiku plant carbohydrates were supplied from local starch in rhizomes (*aruhe*) of fire-encouraged fern, fructan in stems and roots (*kāuru*) of managed *tī kōuka*, and imported *kao* and other preserved foods potentially (e.g. p. 126–30, 144–45 in [30], p. 33–34 in [33]; p. 67 in [57]) [59,60]. At the level of political economy the ethnohistorical record for Murihiku identifies a post-1650 society with high chiefs (*ariki*, from PPN *qariki) and district chiefs (*rakatira*) who managed local native resources and foods distributed through exchange (*kaihaukai*) networks and events (p. 91–103, 126–30 in [30]) [16,33].

In a compounding or alternative variable, hypothetical southern *I*. *batatas* distribution may have been affected by sociopolitical change that impacted exchange networks, perhaps associated with migration and enhanced competition and territoriality from ~1650 CE in particular [30] (p. 26–37 in [33]). This variable could be assessed locally and beyond in comparative archaeological and oral traditional research focused on changing Māori economics and politics (including politics of the defended *pā*), especially between 1500–1800 CE. For example, in one locality of northeastern Te Ika-a-Māui, Māori agricultural abandonment by the later 18th century can be linked to regional traditions of political instability and the threat of warfare [54]. But in far northern and offshore northeastern Te Ika-a-Māui, the abandonment of temperature-sensitive, wetland *C*. *esculent*a (i.e. wet *taro*) production after 1500 CE is broadly coincident with, and related more plausibly to, the LIA onset [15,76]. Any assessment of the southern loss model will need to consider the nature and timing of these political and possible climate change impacts in Aotearoa.

Qualitative and quantitative tests are proposed.

- Further archaeological investigations at Pūrākaunui and Waikouaiti River mouth to investigate the presence and duration of proposed Māori production soil and crop store use.
- New AMS chronologies from paleoclimate proxies [73] and sediment, palynological and other botanical studies from dated environmental archives (e.g. [78]) to evaluate climate and plant management change in Murihiku.
- Traditional and archaeological research and chronological modeling to test the correlation between climate proxies and sociopolitical change across Aotearoa.

## Conclusion

Credible evidence that *rua kūmara* stores were constructed and used at Pūrākaunui between 1430 and 1460 CE contributes a pre-Columbian *I*. *batatas* datum from an early southern Polynesian settlement margin that has been unrecognized previously. We propose that the introduction of this technology to Pūrākaunui in the earlier 15th century from a warmer Aotearoa region may have been stimulated by local socioeconomic impacts of megafaunal loss. It is possible also that management of these *rua kūmara* contributed to the early political economy origins or maintenance of Polynesia’s southernmost residential chiefdom. Certainly, the discovery of *rua kūmara* along a coastline associated with powerful traditional chiefs and enigmatic oral traditions of *kūmara* presence otherwise connects archaeology and *mātauranga* in space at least.

We have suggested also that Otago *rua kūmara* were abandoned largely because of the impacts of the Southern Hemisphere LIA or sociopolitical disruption, if not a combination of both. Whatever the precise reason(s), we propose that this abandonment accounts for an emphasis on loss in southern *kūmara* traditions beyond a single, warm, and well-defended coastal Otago locality (Waikouaiti). And, as we have defined theses of change for empirical testing, our hope is to have modeled respect as much as science in the engagement of Māori knowledge and archaeology to improve understanding of Te Waipounamu’s rich cultural past.

## Supporting information

S1 Fig Māori archaeological midden site no. I44/21 at Pūrākaunui. (Upper.) Contour map from mean sea level (msl) datum locating I44/21 S pit complex (Figs 1 and 2) and the northern anthropic soil profile (S5A Fig), after map data sourced from LINZ (Land Information New Zealand) Data Service licensed for reuse under CC BY 4.0. (Lower.) View from I44/21 S pit complex, about 7m above msl (Fig 1), looking west across the tidal inlet channel. The northeast oriented Āraiteuru coastline is visible in the background.
(TIF)

S2 Fig Reference and archaeobotanical (I44/21 S) specimens. (A) SEM images of *T*. *orientalis* stem specimens presenting comparable vascular structures. (Left.) Carbonized archaeological stem fragment identified as *T*. *orientalis* and dated as Wk-38601, from excavated I44/21 S pit base (P3). This is the only herb specimen identified among macrobotanical remains recovered from I44/21 S [41]. (Right.) Reference *T*. *orientalis* stem specimen collected live from central Otago, 2015. Images courtesy of Otago Micro and Nanoscale Imaging, UO. Archaeological starch granule mass from P3 posthole fill, unit H8, I44/21 S, with birefringent granules presenting centric crosses in polarized light. Individually resolved circular granules of this mass appear >5 μm length in the main, so it is unlikely to be from *C*. *esculenta* corm (S3 Table). Aggregates or ‘clumps’ of granules in various individual size ranges >5 μm are described otherwise from *I*. *batatas* roots (Figs 7m, 7n, 8j and 8k in [48], p. 57 in [50]), *L*. *siceraria* rinds (p. 69 in [50]), and *C*. *laevigatus* seeds (p. 203, Fig 9 in [53]). Among these, *I*. *batatas* may present granule masses enclosed in “a single external package” of “variable size, shape and number” with centric crosses in polarization (p. 57 in [50]), as in the mass image.
(EPS)

S3 Fig Maximum length of single starch granules (‘specimens’), I44/21 S. Specimens from P4 base, unit K5 (x2 granules at 9.8 and 15.7 μm) and P3 posthole, H8 (x34 granules, 6.4–24.2 μm inclusive). Horizontal reference line is at the 13.2 μm threshold discussed in text.
(TIF)

S4 Fig Experimental research results for *Ipomoea batata*s in Dunedin. (Left image.) *I*. *batata*s plant with multiple tuberous roots (generally 2–3 cm wide) grown by IGB in Dunedin from November 2019, harvested and photographed May 2020. This plant was grown under cover in a friable, potted soil (18 cm rim diameter) of low fertility. The relatively thin but starchy tuberous roots may be compared with those of an earlier 19th century Te Ika-a-Māui *kūmara* variety described as “the size and shape of the finger, and extremely farinaceous and nutritious” (p. 114 in [14]). Larger and heavier tuberous *kūmara* roots have been grown outside in a 21st century Dunedin garden as well [72]. Scale bar increments 1.0 cm. (Right image.) *I*. *batata*s roots transported live to Dunedin from central Te Ika-a-Māui in Spring 2017. The roots were dried over summer 2017–2018 and stored by IGB (cf. *kao*) in an unheated room until October 2020 with no measurable loss of condition (photographed October 2020). Live, tuberous *I*. *batata*s roots stored from Autumn in this location succumbed to fungal rot and died during colder winter months of the same year.
(TIFF)

S5 Fig Mixed archaeological soil and rounded depressions in profile from I44/21, Pūrākaunui, cf. pre-1769 mixed Māori cultivation soil profiles with basal depressions (proposed planting pits) from Tata Beach, Ligar Bay and Triangle Flat, northwestern Te Waipounamu. (A) Pūrākaunui channel scarp >4m above high tide exposed briefly during erosion in 2007. Below surface aeolian sand (L1) 1-2m thick, a dark, mixed sandy soil 20-35cm thick presents with scattered, archaeological mollusc valves, charcoal fragments and stones and two small basal depressions (‘feature(s)’, about 40cm and 30cm wide respectively) (S1 Fig). (B) Tata Beach and (C) Ligar Bay dune profiles, including mixed sandy soils with dark sand fill incorporating scattered archaeological molluscs and charcoal fragments and small, basal depressions (30-70cm wide) extending into lighter sand [6,21]. (D) Triangle Flat excavation profile, where a depression about 70cm wide with black sand fill and basal root mold extends from a mixed sandy soil into a marine shell chenier. This pit feature is part of a complex from which starch granules with *I*. *batatas* characteristics have been identified [6,47,74]. Scale bar is 10 cm in each panel.
(TIF)

S6 Fig (TIFF)

S1 Table Stratified layers and archaeological features imaged at Fig 3, correlated to Bayesian model phases where applicable, I44/21 S (Fig 5, S2 Table). (DOCX)

S2 Table AMS and LSC (standard) ^14^C ages from atmospheric (A) and marine (M) reservoirs with Bayesian model boundaries and calibrated (cal AD) ages at 95% probability, I44/21 S (by Fig 5, S1 Table, S2 Text). (DOCX)

S3 Table Starch granule attributes of plant parts that may have been used at 15th century Pūrākaunui (after [9,12,14,16,30,46–54,57–60]). (DOCX)

S1 Text Traditional *kūmara* sources: Description and exegesis. (DOCX)

S2 Text Local marine ΔR and Bayesian modeling with outlier analysis for ^14^C ages from I44/21. (DOCX)
