# Supplementary figures and images for: Archaeological science meets Māori knowledge to model pre-Columbian sweet potato (Ipomoea batatas) dispersal to Polynesia’s southernmost habitable margins
Source: PLoS One. 2021 Apr 14;16(4):e0247643. doi: 10.1371/journal.pone.0247643 (PMC8046222; doi:10.1371/journal.pone.0247643)

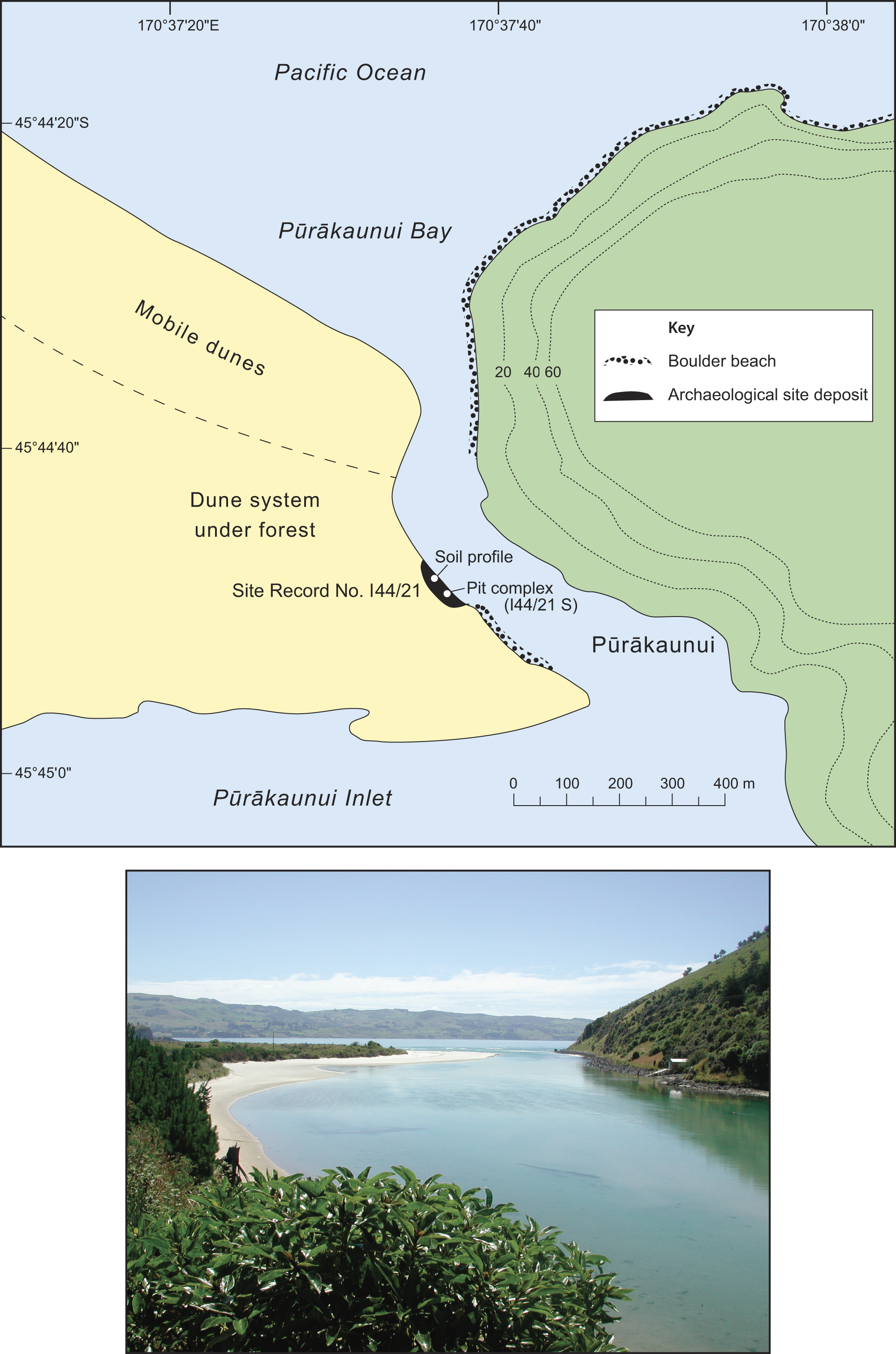

Supplement: S1 Fig — (Upper.) Contour map from mean sea level (msl) datum locating I44/21 S pit complex (Figs 1 and 2) and the northern anthropic soil profile (S5A Fig), after map data sourced from LINZ (Land Information New Zealand) Data Service licensed for reuse under CC BY 4.0. (Lower.) View from I44/21 S pit complex, about 7m above msl (Fig 1), looking west across the tidal inlet channel. The northeast oriented Āraiteuru coastline is visible in the background. (TIF) [file pone.0247643.s001.tif]

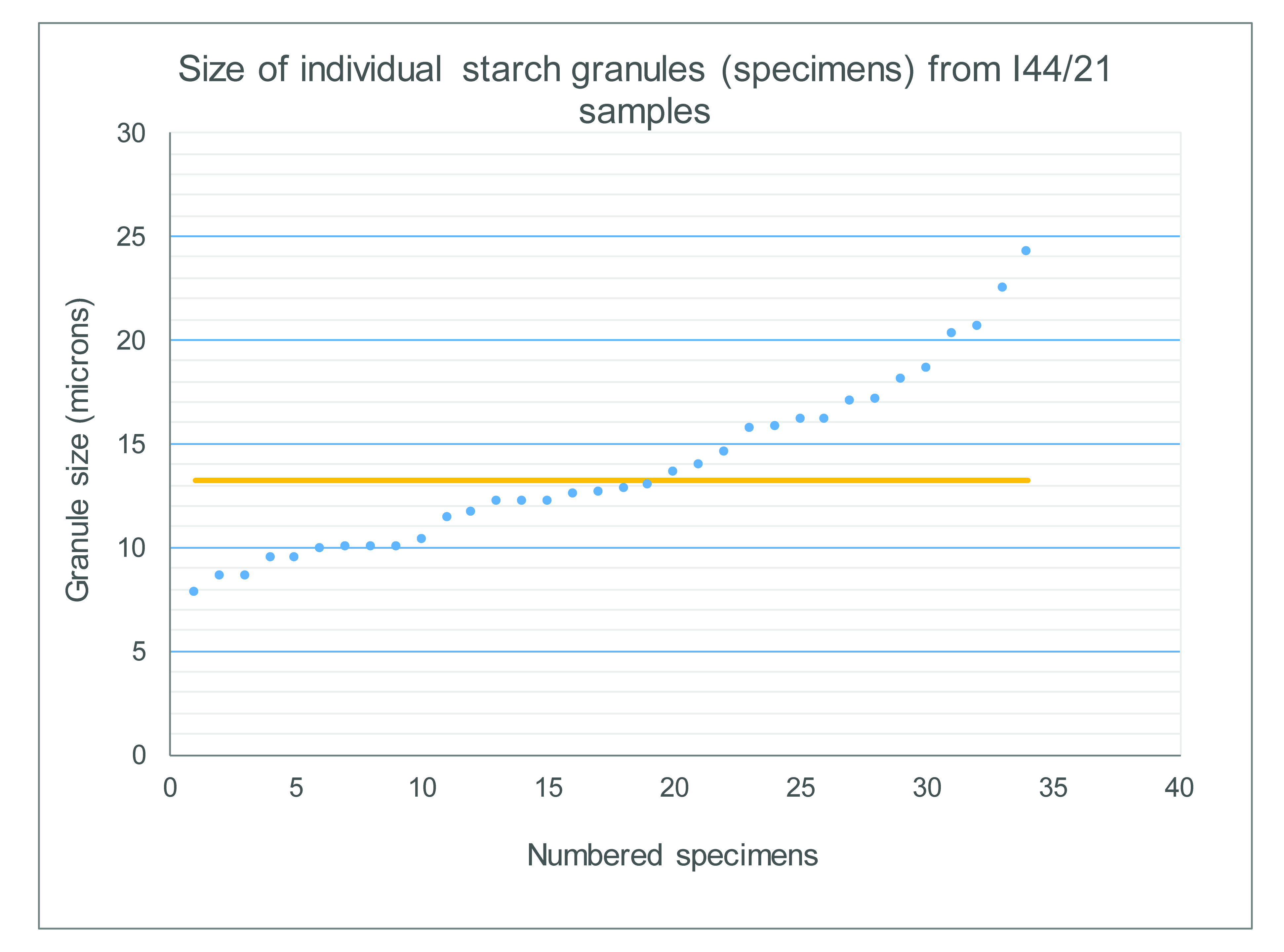

Supplement: S3 Fig — Specimens from P4 base, unit K5 (x2 granules at 9.8 and 15.7 μm) and P3 posthole, H8 (x34 granules, 6.4–24.2 μm inclusive). Horizontal reference line is at the 13.2 μm threshold discussed in text. (TIF) [file pone.0247643.s003.tif]

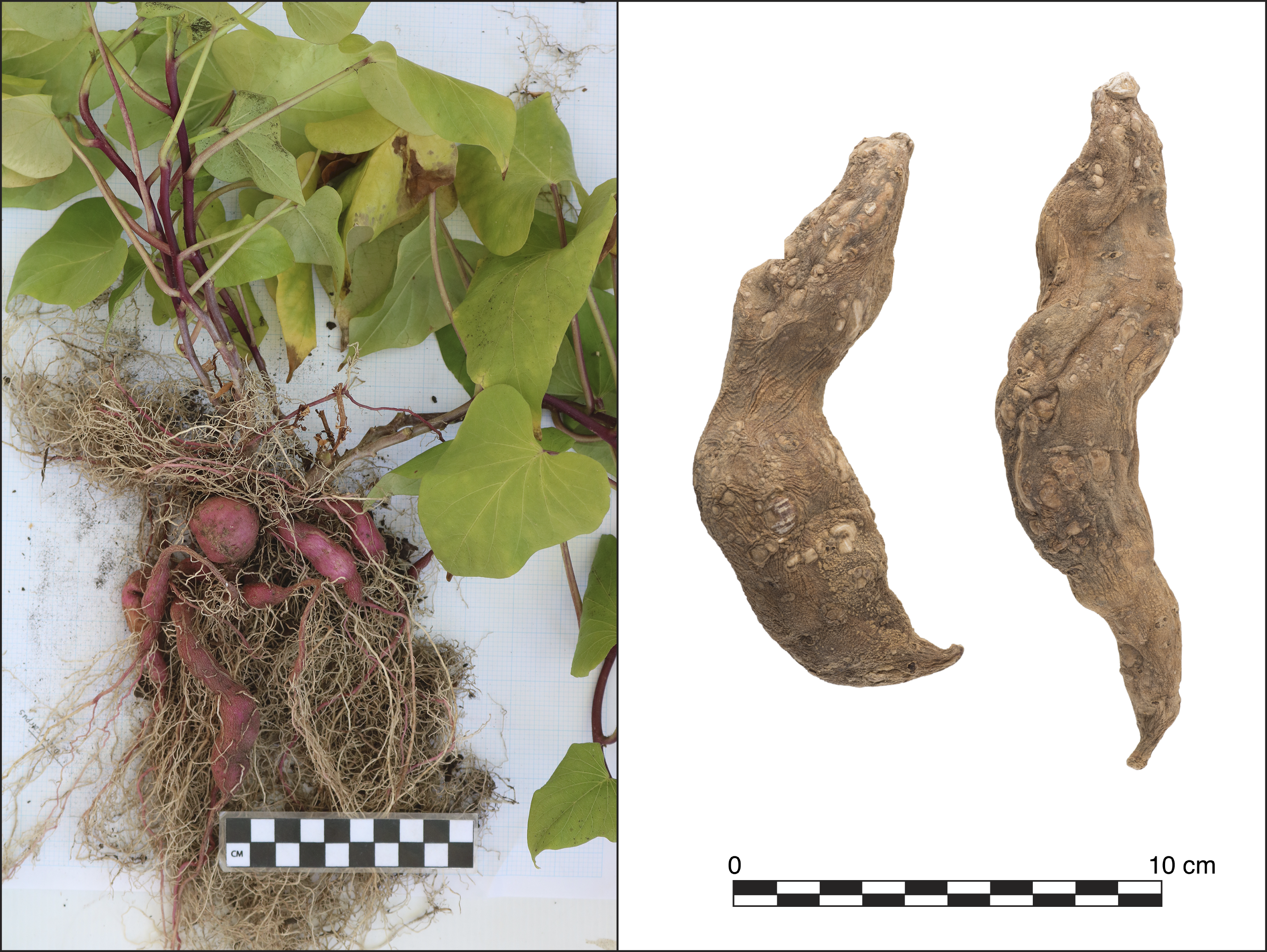

Supplement: S4 Fig — (Left image.) I. batatas plant with multiple tuberous roots (generally 2–3 cm wide) grown by IGB in Dunedin from November 2019, harvested and photographed May 2020. This plant was grown under cover in a friable, potted soil (18 cm rim diameter) of low fertility. The relatively thin but starchy tuberous roots may be compared with those of an earlier 19th century Te Ika-a-Māui kūmara variety described as “the size and shape of the finger, and extremely farinaceous and nutritious” (p. 114 in [14]). Larger and heavier tuberous kūmara roots have been grown outside in a 21st century Dunedin garden as well [72]. Scale bar increments 1.0 cm. (Right image.) I. batatas roots transported live to Dunedin from central Te Ika-a-Māui in Spring 2017. The roots were dried over summer 2017–2018 and stored by IGB (cf. kao) in an unheated room until October 2020 with no measurable loss of condition (photographed October 2020). Live, tuberous I. batatas roots stored from Autumn in this location succumbed to fungal rot and died during colder winter months of the same year. (TIFF) [file pone.0247643.s004.tiff]

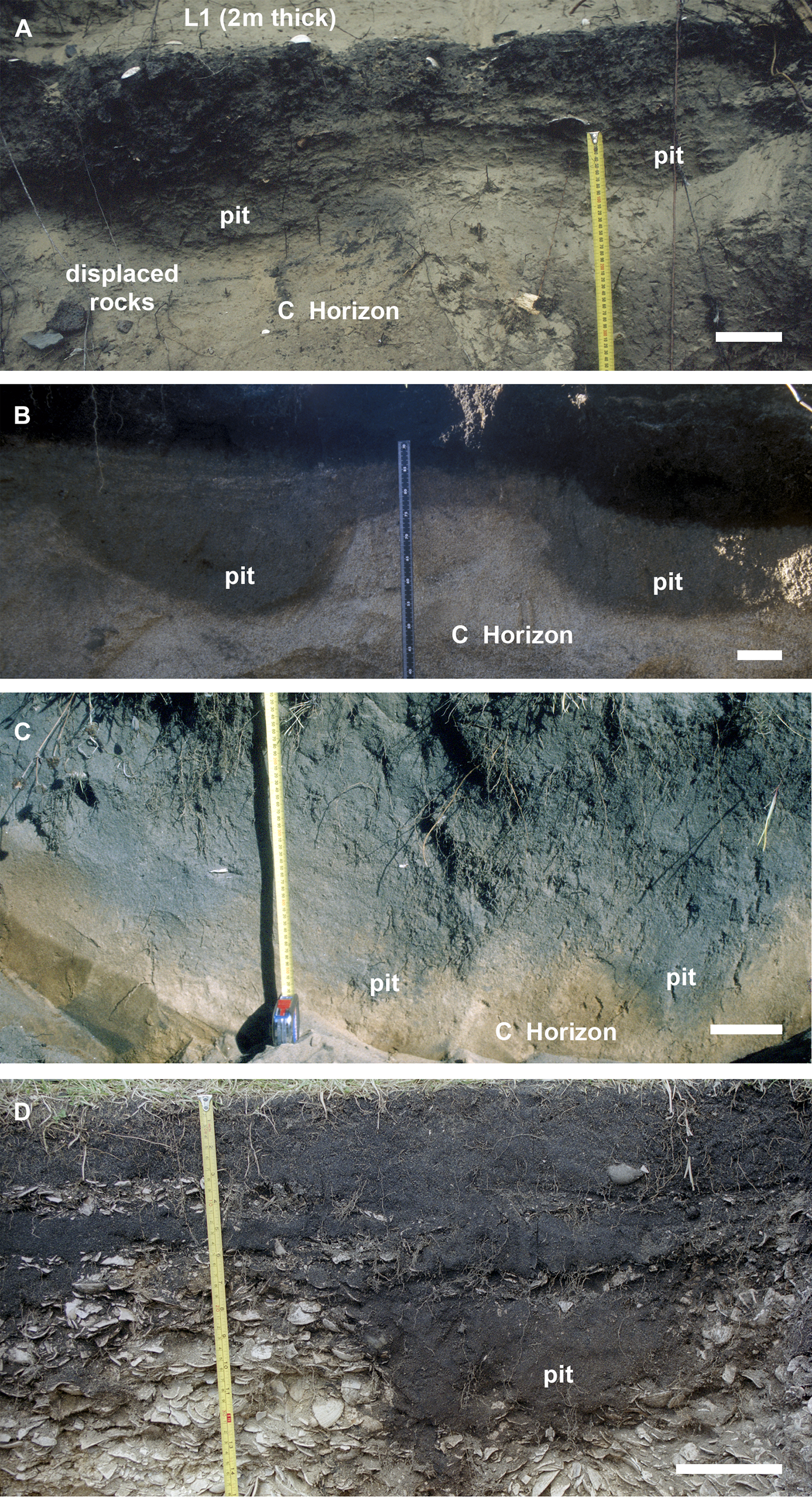

Supplement: S5 Fig — (A) Pūrākaunui channel scarp >4m above high tide exposed briefly during erosion in 2007. Below surface aeolian sand (L1) 1-2m thick, a dark, mixed sandy soil 20-35cm thick presents with scattered, archaeological mollusc valves, charcoal fragments and stones and two small basal depressions (‘feature(s)’, about 40cm and 30cm wide respectively) (S1 Fig). (B) Tata Beach and (C) Ligar Bay dune profiles, including mixed sandy soils with dark sand fill incorporating scattered archaeological molluscs and charcoal fragments and small, basal depressions (30-70cm wide) extending into lighter sand [6,21]. (D) Triangle Flat excavation profile, where a depression about 70cm wide with black sand fill and basal root mold extends from a mixed sandy soil into a marine shell chenier. This pit feature is part of a complex from which starch granules with I. batatas characteristics have been identified [6,47,74]. Scale bar is 10 cm in each panel. (TIF) [file pone.0247643.s005.tif]

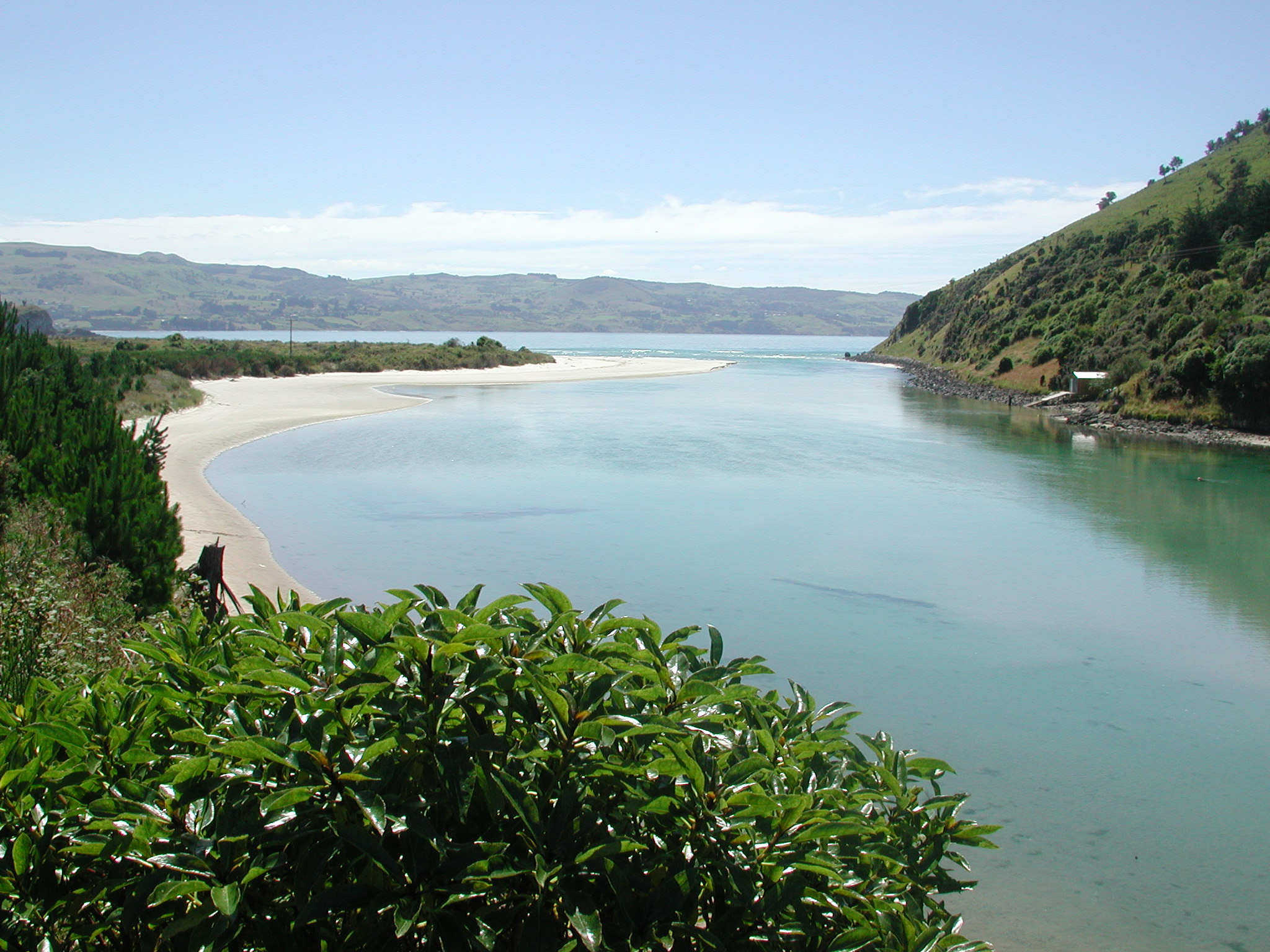

Supplement: S6 Fig — (TIFF) [file pone.0247643.s006.tiff]
