# Supplementary material for: Archaeological science meets Māori knowledge to model pre-Columbian sweet potato (Ipomoea batatas) dispersal to Polynesia’s southernmost habitable margins
Source: PLoS One. 2021 Apr 14;16(4):e0247643. doi: 10.1371/journal.pone.0247643 (PMC8046222; doi:10.1371/journal.pone.0247643)
Supplement: S1 Table — (DOCX) [file pone.0247643.s007.docx]

**S1 Table.** Stratified layers and archaeological features imaged at Fig 3, correlated to Bayesian model phases where applicable, I44/21 S (Fig 5, S2 Table).

| **L(ayer) # F(eature):** thickness (maximum) | **Bayesian model phase** | **General description including basic matrix colors by Munsell system (specification on damp earth)** |
| --- | --- | --- |
| **L1:**  >1000 mm | _ | Natural, light yellowish brown (2.5Y 6/3) sand, post-19th century deposition (aeolian). |
| **L2:**  200 mm (non-fill) | **L2** | Cultural, variable dark gray (10YR 4/1-2.5Y 4/1), dark grayish brown (10YR 4/2) or very dark gray (10YR 3/1-2.5Y 3/1) to/or black (10YR 2/1-2.5Y 5/1) sand incorporating scattered midden components and discrete primary midden deposits, extending over and into fill of pits (below) where depth may be >200mm. Midden sediments are primarily mollusc bivalves, some articulated finfish head parts and vertebrae (some articulated)), angular woody charcoal, oven stone fragments and in certain deposits (including pit fill), many burnt mollusc valves.^a^ |
| **L2u[pper]:**  200 mm (non-fill) | **L2u** | As for L2 above but upper aspect^b^ to or just below L1/L2 border incorporating post-1690 native plant charcoal (cf. NZA 62297, Table S2) and intrusive 19th century material culture (e.g. clay, glass) and fauna. |
| **L3:**  250 mm | _ | Natural (aeolian) to cultural, light yellowish brown (2.5Y 6/3) to/or dark to very dark grayish brown (2.5Y 4/2-3/2) sand with scattered midden components only (as per L2).^a^ |
| **L4:**  200 mm | **L4** | Cultural, dark grayish brown (10YR 4/2) to very dark grayish brown (10YR 3/1 to/or 2.5Y 3/1) sand with scattered midden components as well as discrete primary midden deposits (as per L2). |
| **L5:**  >500 mm |  | Natural (aeolian C-horizon), brown (10YR 4/3) grading into light yellowish brown (2.5Y 6/3) sand. Some upper level cultural intrusion (angular charcoal, isolated mollusc bivalves) or leaching. |
| **Pit:**  700 mm (cap to fill) | **P3cap/3**^c^  **P3fill**^c^ | Quadrangular excavation >2 m long with straight sides, sometimes stepped, and posthole remains (internal and external) of covering superstructure, above or cut through L4. Colors of dominant sand fill as for and from L2 (above) but also including light olive brown (2.5Y 5/3) sand lenses. Fill includes scattered midden and discrete, generally oblique midden deposits with articulated bivalves originating from L2. Among numbered excavated pits, P3 is capped by midden lenses. |
| **Oven:**  400 mm | - | Cultural, rounded, excavated depression (earth oven, *umu*) <2 m long cut from L2 and L4, but especially characteristic of L4. Fill is predominantly very dark grayish brown (10YR 3/1) sand, angular (woody) charcoal and (sometimes) *in situ* cooking stones. |

^a^ Molluscs are predominantly soft shore bivalves *Paphies australis* Gmelin, 1790 and *Austrovenus stutchburyi* (Wood 1828)*,* gastropod *Amphibola crenata* (Gmelin, 1791), and hard shore bivalve *Mytilus edulis* Linnaeus, 1758 with visually notable minor representation of large gastropod *Haliotis* spp. Finfish by bones, teeth and otoliths are predominantly inshore marine *Pseudophycis bachus* (J. R. Foster, 1801) and *Thyrsites atun* (Euphrasén, 1791). Among minor midden components, teeth and bones of domestic *Canis familiaris* (often modified), commensal *Rattus exulans* (Peale 1848), and bones of smaller, predominantly coastal or marine avifauna, are reported in all deposits. Remains of large, flightless, terrestrial *moa* ratites (Aves: Dinornithiformes) are more scant but in all deposits also as long bone fragments principally that have been modified profoundly in reduction [37-39]. Sea mammal remains (largely Pinnipedia) are rare.

^b^ The upper aspect is equivalent generally to earlier I44/21 unit designation L2a [38].

^c^ Representing basal pit context only.
