# Supplementary material for: Archaeological science meets Māori knowledge to model pre-Columbian sweet potato (Ipomoea batatas) dispersal to Polynesia’s southernmost habitable margins
Source: PLoS One. 2021 Apr 14;16(4):e0247643. doi: 10.1371/journal.pone.0247643 (PMC8046222; doi:10.1371/journal.pone.0247643)
Supplement: S2 Table — (DOCX) [file pone.0247643.s008.docx]

S2 Table. AMS and LSC (standard) ^14^C ages from atmospheric (A) and marine (M) reservoirs with Bayesian model boundaries and calibrated (cal AD) ages at 95% probability, I44/21 S (by Fig 5, S1 Table, S2 Text).

| **Boundary** or **Context-AMS/LSC**  Reservoir-taxon | Short-life sample^a^ | Lab# | ^14^C age BP | δ^13^C‰  IRMS^b^ | cal AD, unmodeled, prior ^b, c^ | cal AD, modeled **boundary,**  posterior ^b, c^ |
| --- | --- | --- | --- | --- | --- | --- |
| **Boundary End L2u** |  |  |  |  |  | **1445-1494** |
| **L2u**^d^**–AMS** |  |  |  |  |  |  |
| A–Myrtaceae | Yes (twig) | NZA 62297 | 149±17 | -25.9±0.2 | 1692-1950 | - |
| A– *Hebe* sp. | Yes (twig) | NZA 60804 | 393±19 | -25.7±0.2 | 1457-1626 | - |
| M–*P. australis* | Yes | Wk-44570 | 873±22 | - | 1345-1621 | 1445-1481 |
| M–*stutchburyi* | Yes | Wk-44569 | 911±20 | - | 1312-1570 | 1445-1480 |
| **L2**^d^ **– LSC** |  |  |  |  |  |  |
| M–*P. australis* | Unknown | Wk-14099 | 868±35 | 1.7±0.2 | 1343-1632 | 1445-1463 |
| **L2**^d^**–AMS** |  |  |  |  |  |  |
| M–*P. australis* | Yes | Wk-44572 | 896±20 | - | 1323-1591 | 1445-1463 |
| M–*P. australis* | Yes | Wk-44571 | 862±20 | - | 1356-1635 | 1445-1463 |
| A–*Melicytus* sp. | Yes (twig) | Wk-37505 | 488±22 | -27.3±0.2 | 1421-1477 | 1445-1460 |
| A–*Pseudopanax* sp^.^ | Unknown | Wk-37504 | 492±22 | -22.6±0.2 | 1420-1460 | 1445-1460 |
| A–*Melicytus* sp^.^ | Yes (twig) | NZA 62299 | 435±17 | -26.4±0.2 | 1450-1615 | 1445-1464 |
| **Boundary P3 cap/L2** |  |  |  |  |  | **1443-1459** |
| **P3 cap–AMS** |  |  |  |  |  |  |
| M–*P. australis* | Yes | Wk-37520 | 889±20 | 1.6±0.2 | 1329-1600 | 1443-1459 |
| **P3 fill–LSC** |  |  |  |  |  |  |
| M–*P. australis* | Unknown | Wk-12597 | 897±34 | 1.6±0.2 | 1315-1602 | 1441-1458 |
| M–*P. australis* | Unknown | Wk-12596 | 958±35 | 1.8±0.2 | 1285-1525 | 1441-1458 |
| **P3 fill–AMS** |  |  |  |  |  |  |
| M–*P. australis* | Yes | Wk-44573 | 879±21 | - | 1338-1614 | 1441-1458 |
| M–*P. australis* | Yes | Wk-37522 | 907±20 | 1.5±0.2 | 1315-1576 | 1441-1458 |
| M–*P. australis* | Yes | Wk-37521 | 927±21 | 1.1±0.2 | 1305-1546 | 1441-1458 |
| A–*T. orientalis* | Yes (stem) | Wk-38601 | 485±20 | - | 1426-1477 | 1441-1458 |
| **Boundary L4/P3 fill** |  |  |  |  |  | **1440-1456** |
| **L4–LSC** |  |  |  |  |  |  |
| M–*P. australis* | Unknown | Wk-14100 | 930±35 | 1.5±0.2 | 1296-1559 | 1431-1455 |
| **L4–AMS** |  |  |  |  |  |  |
| M–*A. stutchburyi* | Yes | Wk-44574 | 886±22 | - | 1331-1608 | 1431-1455 |
| A–*Pseudopanax* sp. | Unknown | Wk-37503 | 521±20 | -27.1±0.2 | 1415-1452 | 1432-1455 |
| A–Myrtaceae | Yes (twig) | Wk-37502 | 542±21 | -28.1±0.2 | 1406-1446 | 1431-1455 |
| A–*Melicytus* sp. | Unknown ^e^ | Wk-37501 | 655±20 | -27.3±0.2 | 1301-1400 | 1431-1455 |
| A– Myrtaceae | Yes (twig) | NZA 62298 | 437±18 | -28.1±0.2 | 1450-1615 | 1435-1455 |
| A–*Hebe* sp. | Yes (twig) | NZA 60803 | 473±19 | -25.5±0.2 | 1430-1485 | 1435-1455 |
| **Boundary Start L4** |  |  |  |  |  | **1425-1455** |

^a^ Short-life samples <10 ^14^C years at death include terrestrial plant twigs or branchlets (both lumped as ‘twig’ in table), *T. orientalis* (rush) stem (‘stem’ in table), and estuarine mollusc valve edges as discussed in the main article.

^b.^ Dash (-) indicates that δ^13^C value is not reported for IRMS, or ^14^C age is not modelled, as applicable.

^c.^ Atmospheric calibrations by SHCal20 [43] and marine calibrations by Marine20 curve [44] using local weighted mean ΔR -162±28, with posterior chronology by Bayesian age model in OxCal v. 4.4 (Fig 5, S2 Text).

^d^ Above large pits P3 and P4 by stratigraphic superposition.

e. In context it is suspected that this determination has been affected by inbuilt age (S2 Text), even though its short-life status cannot be assessed botanically (hence ‘unknown’ in table).
