# Supplementary material for: Archaeological science meets Māori knowledge to model pre-Columbian sweet potato (Ipomoea batatas) dispersal to Polynesia’s southernmost habitable margins
Source: PLoS One. 2021 Apr 14;16(4):e0247643. doi: 10.1371/journal.pone.0247643 (PMC8046222; doi:10.1371/journal.pone.0247643)
Supplement: S3 Table — (DOCX) [file pone.0247643.s009.docx]

**S3 Table.** Starch granule attributes of plant parts that may have been used at 15th century Pūrākaunui (after [9, 12, 14, 16, 30, 46-54, 58-61]).

| **Category** Taxon, Māori name | Source | N^a^ | Length (μm) | Shape, 2D | Cavity or fissures at hilum (shape)^b^ |
| --- | --- | --- | --- | --- | --- |
| **Polynesian crop** |  |  |  |  |  |
| *Colocasia esculenta*, *taro*^c^ | Corm | 100 | <1.0-12.0^a^ | Polygonal, circular, faceted | No(?)^b^ |
| *Lagenaria siceraria, hue* ^c^ | Rind^d^ | - | 8.5-25 | “Spherical and oval, sometimes faceted” ^e^ | Yes (round)^e^ |
| *Ipomoea batatas*, *kūmara* | Root | 100s | 2.5-55 | Polygonal, circular, semicircular, faceted | Yes (var. shapes, fissures)^f^ |
| **Aotearoa native** |  |  |  |  |  |
| *Calystegia sepium*, *pōhue* | Root | 100 | 3.2-19.4 | Circular, semicircular to oval | No |
| *Corynocarpus laevigatus*, *kōpīa* | Seed | 398 | 2.2-13.2 | Polygonal, circular, semicircular, faceted | Yes (round, radial, stellate fissures)^g^ |
| *Microtis* spp., *māikaika* | Tuber | 100 | 6.4-51.5 | Oval-elongate oval | No |
| *Pteridium esculentum*, *aruhe* | Rhizome | 100 | 8.9-32.8 | Oval, teardrop | No |
| *Typha orientalis*, *raupō* | Rhizome | 100 | 5.8-28.2 | Circular to oval, irregular circular | No |

^a^ N.= minimum number of granules measured, including table 1, fig 11 in [53]. The *C. esculentum* length range acknowledges fig 5 in [47], although reference granules are usually <6 μm, especially in mass (p. 205 and references in [53]; see also [9, 47, 48, 51, 52]). For *I. batatas*, ‘100s’ follows p. 57 in [50], and [9, 46-54] where applicable.

^b^ Hila of native plants are described from starch granules of vouched, contemporary reference specimens [46, 53]. Cavity assessment was indeterminate generally for the small (<6 μm) reference *C. esculentum* starch granules examined in 2020 light microscopy work.

^c^ Inclusion recognizes the historical claim that *taro* and *hue* were exchanged to Murihiku traditionally by “the system of *kaihaukai*” (reciprocal feasting) (p. 67 in [58]). Otherwise, Otago production of these plants is not supported on climatic or any known archaeological, experimental or traditional grounds.

^d^ *L. siceraria* pulp granules would have been removed before the exocarp was dried for storage and exchange, and there is no ethnobotanical evidence that Māori stored *L. siceraria* seeds as food (p. 244-55 in [14], p. 303 in [16]). Moreover, the hilum in *L. siceraria* seed and pulp starch granules is “not visible” (p. 69 in [50]). Accordingly, the rind granule only is considered relevant for the purposes of characterization in Otago.

^e^ After description of a “distinct hilum” in “single grains” of the rind as a “clear spot with a round cavity”. The granule presents radiating fissures “sometimes” (p. 69 in [50]).

^f^ Cavities at hila of reference *I. batatas* roots vary in shape between round and nonround or asymmetric (S3 Figure). Fissures at hila are sometimes “crossed, winged- to y-shaped, or star-shaped” (figs 2a, 4a in [46]; see also Fig 6 in article, figs 8e-g, 9e-g in [51], fig 7e in [52]). From a UO reference set of 65 *I. batatas* granules >13.2 μm, cavities at hila present in 40 specimens (62%, n=65), over half of which are nonround in shape, including fissured hila.

^g^ For a “majority” of processed reference *C. laevigatus* granules, “a distinct round, centric hilum … is visible as a small central cavity … sometimes with radial or stellate fissures” (p. 203 in [53]).
