# Supplementary material for: Archaeological science meets Māori knowledge to model pre-Columbian sweet potato (Ipomoea batatas) dispersal to Polynesia’s southernmost habitable margins
Source: PLoS One. 2021 Apr 14;16(4):e0247643. doi: 10.1371/journal.pone.0247643 (PMC8046222; doi:10.1371/journal.pone.0247643)
Supplement: S1 Text — (DOCX) [file pone.0247643.s010.docx]

Supplementary 1 Text for:

**Archaeological science meets Māori knowledge to model pre-Columbian sweet potato (*Ipomoea batatas*) dispersal to Polynesia’s southernmost habitable margins**

Ian G. Barber^1*^, Thomas F. G. Higham^2^

^1^ Archaeology Programme, School of Social Sciences, Division of Humanities, University of Otago, Dunedin, New Zealand

^2^ Oxford Radiocarbon Accelerator Unit, Research Laboratory for Archaeology and the History of Art, School of Archaeology, University of Oxford, Oxford, United Kingdom

* Corresponding author: ian.barber@otago.ac.nz (IGB)

**Traditional *kūmara* sources: description and exegesis**

**Introduction and method**

In the main article, archaeological science is evaluated alongside published Māori *kūmara* (*Ipomoea batatas*) traditions and knowledge (*mātauranga*) for Murihiku (southern South Island/Te Waipounamu) localities (Fig 1). These Māori historical traditions and *mātauranga* have been published from and since the earlier 19th century. The relevant body of lore now incorporates etiological myth, historical narrative and *whakapapa* (genealogies) for a succession of northern lineages who relocated to live among earlier Te Waipounamu *iwi* (tribes) [16, 22-33]. Its published record is analyzed in this supplementary text in accordance with critical-text methods of exegesis that have been applied generally in Māori scholarship [28-36, 79]. Our purpose is to explain how and why southern *kūmara* lore elements are accepted as authentic ancient traditions for analysis in this study.

Critical-text analysis of Māori historical records must allow that these traditions were generated and transmitted first by oral means only before the 19th century. This introduces some interpretive uncertainty, complicated by the reality that “multiple accounts of one event are the staple of tribal narratives,” as Ngāi Tahu scholars Tau and Anderson observe (p. 17 in [33]). Variation may have occurred as the result of recitation error, or deliberate amendment for political or other reasons, potentially affecting the transmission of documentary records also. In some situations one might be able to investigate historical variations of a tradition through careful comparative analysis in context (e.g. 135-82 in [32]), but this may not be conclusive or possible even. A further issue for the extant traditional record is that *tohunga* (expert, specialist) or other custodians of *iwi* tradition may have withheld certain oral traditions from outsiders, especially if the knowledge was considered *tapu* (prohibited, set apart, sacred) (e.g. p. 10, 16-17 in [32]).

In our analysis, thematic replication across independent sources is the primary threshold for acceptance, and we do not assume that the available documentary record is complete. The status and intention of the recorder in context is important also, and considered here first.

**Source assessment**

Ngāi Tahu (southern var. Kāi Tahu) with its east coast Te Ika-a-Māui origins is the most recent *iwi* arrival of record in southern Te Waipounamu [30-33]. Today the governance authority Te Rūnanga o (Council of) Ngāi Tahu represents the Indigenous lineages of Te Waipounamu formally beyond the northern end of the island [80]. In 1844, Edward Shortland as “Protector of Aborigines” of the then New Zealand British colony described Ngāi Tahu traditions as “more true and correct” than those of any other *iwi* (p. 25 in [30]). Multiple and carefully preserved Ngāi Tahu *whakapapa* (genealogical histories) were compiled between 1880 and 1920 in private tribal settings also, rather than in the sometimes tendentious hearings of the contemporary Native Land Court (p. 33-35 in [32]).

Other Ngāi Tahu traditions were collected in various cultural-historical settings, beginning with the earlier 19th century accounts of missionary J. F. H. Wohlers from Ruapuke, an offshore island of southern Murihiku where Te Waipounamu authorities had relocated (Fig 1, p. xi in [31]). Later 19th century European collectors of Ngāi Tahu traditions include government officials Edward Shortland (as above) and John White, the last employed by the Crown for ethnographic work specifically, and missionary Canon James Stack who was motivated to convert, but also, understand and document [22-27, 30-32, 79].

Important ethnohistorical materials were recorded in the early 20th century by journalists and researchers James Herries Beattie and Hugh Carrington in accordance with contemporary rescue ethnology imperatives. Beattie’s informants included Murihiku sources and Canterbury Ngāi Tahu authority Teone Taare Tikao (1850-1927) in the first decades of the 20th century [16, 29], while Carrington composed a history that drew on a number of Ngāi Tahu authorities in the early 1930s [33]. These materials were compiled while Te Waipounamu community grievances against the Crown for unresolved treaty beaches and injustices were heightened and ongoing [80], and some families of earlier Waitaha and Ngāti Māmoe (southern var. Kāti Māmoe) descent were dissatisfied with the Ngāi Tahu Claim (p. 222 in [32]).

More recently, Ngāi Tahu researcher Rawiri Te Maire Tau has compiled an *iwi* history in which published Ngāi Tahu sources are supplemented by private family records, including original *whakapapa* and other manuscripts from authorities that Beattie had copied or relied on, some predating 1925 (p. 15-31 in [32]). Tau’s study critiques interpretations of Stack and White, evaluates traditional sources comparatively, and provides important insider analysis of primary sources from a research period that tracked the Ngāi Tahu treaty settlement process [32, 80]. Of note, Tau justifies the publication of private family records “because I have seen over the last decade an explosion of utter nonsense” (p. 10 in [32]). This work may be complemented by Ngāi Tahu archaeologist Atholl Anderson’s well-regarded ethnohistorical study [30] (p. 7 in [33]) and thoughtful editorial commentary on Beattie [16] and, with Tau, on Carrington [33].

Our analysis is focused on thematic support or replication across these sources, authorities, and diverse political contexts, with particular regard to the work of Tau and Anderson [16, 30, 32, 33]. It is organized by four primary, cultural-historical southern *kūmara* themes, numbered hereafter.

**1. Kāhui Roko/Rongo**

In central to southern Te Waipounamu Māori sources, the first mythic peoples are identified by collective Kāhui (cluster, swarm, company) names. Prominent among these are Kāhui Tipua and Kāhui Roko (p. 160-61 in [23], v.24 p. 108, v.27 p. 140-45, v.31 p. 195 in [27], p. 22 in [30], p.175-77, in [32]). In 1887 and 1920s accounts from authorities of Waitaha, the earliest, recognized historical Te Waipounamu *iwi*, Kāhui Tipua and Kāhui Roko came on the ancestral Uruao *waka* (p. 267-73 in [32]). Of particular note, Roko is a southern variant of Rongo, the Māori *atua* (=deity, influential ancestor) of *kūmara* in Aotearoa and agriculture elsewhere in Polynesia (e.g. as Lono) [21] (p. 103, 111-12 in [31], p. 175-77 in [32]). Beattie recorded a further Kāhui Roko account from Canterbury authority Tikao apparently that reinforced the *kūmara* connection: “Kahui-Rongo and Kahui-Matua people grew the kumara in Hawaiki, the former bringing the tuber to New Zealand, the latter remaining in Hawaiki, and it was from the Kahui-Rongo that the Maori obtained the kumara” (v.31 p. 195 in [27]). In these accounts Tau interprets Kāhui Rongo “as the Esteemed Line of Rongo,” a name “that returns us back to the root word Rongo and therefore the connection to *kūmara*” (p. 176 in [32]).

Other sources clarify or refer to further Rongo/Roko identities among the earliest Te Waipounamu peoples. In a 19th century address delivered in Canterbury, Stack referenced a chief among Kāhui Tipua with a Rongo name (Rongo-mai), presumably drawing on a relatively local source (p. 160 in [23]). An early 20th century account from one of Beattie’s Murihiku informants identified Kāhui Roko itself as “another tribe of those ancient times” which was split up into five Roko sections. Beattie added that “it was these Kahui-roko people amongst whom the chief O-roko-i-te-ata from Hawaiki landed and was kindly welcomed” (v.24 p. 108 in [27]). This chief is discussed below as Roko i Tua.

**2. Roko/Rongo i Tua (Ko Rongo-i-tua)**

Māori studies scholar Christine Tremewan observes that all *iwi* in Aotearoa have a *kūmara* introduction myth, including the people of southern Te Waipounamu. Tremewan concentrates on an earlier 19th century Murihiku (Ruapuke) version recorded by missionary Wohlers characterized as “Ko Rongo-i-tua.” The Roko/Rongo name is central to this tradition, as in the Te Waipounamu Kāhui accounts above, which are sometimes linked. Ko Rongo-i-tua is “an important southern version” of *kūmara* origins, Tremewan argues, and “more than this.” In “cryptic form”, Tremewan observes, Ko Rongo-i-tua conveys “instructions about the rituals necessary for planting, cultivating and harvesting this most prestigious food plant” (p. 103, 118 in [31]).

Ko Rongo-i-tua and comparable Ngāi Tahu accounts begin with the introduction of dried *kūmara* (*kao*) to Aotearoa people by Hawaiki visitor Rongo/Roko i Tua (or in one version, as above, “O-roko-i-te-ata”). The accounts are linked thematically and in culture history to Te Ika-a-Māui versions (p. 160-61 in [23], p. 99-103 in [24 v. 3]), v24 p. 108 in [27], p. 68-69, 138-40 in [28], p. 119-20 in [31], p. 174-78 in [32] [66]). In the core myth, Rongo/Roko i Tua was offered local native plant foods by people with collective Kāhui names (as above), or in some versions, the people of early settler Toi. Visitor Rongo/Roko declined the non-agricultural foods and prepared *kao* brought from Hawaiki instead. The local Kāhui people who tasted this sweet preparation were “much pleased,” and they or Rongo/Roko prepared the *waka* (canoes) named Ārai Te Uru, Manuka (in southern accounts) and (or) Horouta (sometimes conflated with the Takitimu *waka*) to travel to Hawaiki to retrieve live *kūmara* (p. 426-28 in [16], p. 161 in [23], p. 105-14 with quote p.106 in [24, v.3], p. 15-18 in [25], v.24 p. 108 in [27], p. 32-46 in [28], p. 61-62, 63-64 in [29], p. 15-16 in [30], p. 98-115 in [31], p. 174-78 in [32]).

In some Te Waipounamu accounts, including those of Beattie’s Murihiku informants, the Kāhui group that Rongo/Roko meets in Aotearoa were themselves Kāhui Roko (v.24 p. 108, v.26 p. 76 in [27]). However, in Wohlers’s Ruapuke account, the locals are Kāhui Tupu, or “the indigenous people” who eat wild foods that issue from Aotearoa (*tupu*, ‘issue, begin’; p. 112 in [31). A wild food name resonates also in the version of Canterbury Ngāi Tahu authority Teone Taare Tikao (1850-1927), for whom the locals are Toi-kai-rākau (food of the forest) who are without cultivated produce and “did not know” the *kūmara* food of Roko i Tua (p. 61-62 in [29]).

The ancient prestige of *kūmara* is recalled in common across these Roko i Tua variants, whether by contrast as Roko meets Kāhui Tupu/Toi-kai-rākau, the people of wild foods, or by eponymous, ancestral reconnection with the Aotearoa people of Kāhui Roko.

**3. Failed or lost *kūmara* from Ārai Te Uru, Horouta and Manuka *waka***

In multiple traditions, the *waka* that travelled back to Hawaiki following Roko i Tua’s visit (above) were successful generally in sourcing live *kūmara*, albeit after conflict with Kāhui peoples of Hawaiki (especially Kāhui Rongo) and various problems with ritual requirements. Several accounts of these returning *waka* follow, some of which are not associated directly with Roko i Tua.

In southern versions, the *waka* Manuka returned to an unspecified Aotearoa location where the *kūmara* plants failed to produce (p. 161 in [23], p.12 in [24 v.3], p. 18 in [25], v.24 p. 108 in [27]). In one account, *kūmara* from Manuka “rotted in the ground” because its crew had neglected the designated *karakia* (ritual incantation) (v.24 p. 108 in [27]). For Canterbury authority Tikao, the *“kumera”* of returning Manuka and Horouta were a “straggly few”, and later *waka* would bring “the better kinds” to Aotearoa (p. 62 in [29]). As documented in the main article, linked themes of *kūmara* disappointment and failure in these Ngāi Tahu Manuka and Horouta traditions contrast with Te Ika-a-Māui accounts of *kūmara* distribution in “abundance” from Horouta as the *waka* arrived in Aotearoa (p. 102-03 in [24 v.3]), p. 132-46 in [28], p. 40 in [64]), [66]). Across northern and southern versions, correct protocol and knowledge were recognized as important for production success. One Te Ika-a-Māui account describes *kūmara* from Horouta that were grown successfully by the woman Hinekauirangi because she knew when to set the plant, unlike her brother’s plot of *kūmara* that did not grow (p. 135 [28]). However, *kūmara* production was uniformly unsuccessful in southern accounts of these *waka* where the emphasis is on ritual failure.

The other *waka* to arrive back from Hawaiki in multiple Ngāi Tahu versions of this tradition was Ārai Te Uru. In some accounts, this *waka* landed *kūmara* in Te Ika-a-Māui and northeast Te Waipounamu locations before ending its voyage in Otago (p. 16 in [30]). From the narratives of Canterbury *tohunga* and an original Moeraki (North Otago) Māori text of 1896, Ārai Te Uru returned to Otago finally with *kūmara* and *hue* (*Lagenaria siceraria*), but only to lose its cargo overboard in stormy seas off Moeraki (p. 61 in [22], p. 161 in [23], p. 204-07 in [28]; p. 15-16 in [30], p. 175 in [32]). In the Moeraki text the agricultural cargo included *taro* (*Colocasia esculenta*) as well (p. 204, 206 in [28]). From this region, Edward Shortland’s 1844 North Otago Māori guides retold the Ārai Te Uru account and identified “globular stones” on the beach less than 10 km south of Moeraki as *kūmara* from the *waka* (p. 15 in [30], see also p. 426, 442 in [16], p. 161 in [23], p. 191 in [24 v.3], p. 18 in [25], p. 204-06 in [28]; p. 63-64 in [29]). Some later 19th-century Te Waipounamu Māori even speculated that the Ārai Te Uru cargo had included wheat seeds based on observations of inclusions in North Otago beach boulders ( p. 426 in [16], p. 64 in [29].)

In all narratives, Ārai Te Uru wrecked finally on the Otago coast of its traditional name to account for various features of the cultural landscape, representing “the links between the cosmological world of the gods and present generations” (Schedule 103 in [80] and narrative details in p. 426, 568 in [16], p. 61 in [22], p. 161 in [23], p. 179 in [24 v.2], p. 18 in [25], v.24 p. 108-09 in [27], p. 204-07 in [28], p. 63-64 in [29], p. 15-16 in [30], p. 114-15 in [31], p. 174-78, 212 in [32]). In some versions the Ārai Te Uru wreck occurred because the crew’s *karakia* or acts were not ritually correct (p. 63 in [29], p. 114-15 in [31]), an explanation that parallels Horouta *waka* accounts of *kūmara* failure in Aotearoa, above.

One reason for the variant Te Ika-a-Māui and Te Waipounamu versions of these origin traditions may be that migrating northern lineages adapted northern Rongo i Tua and associated *waka* accounts to the more marginal agricultural south after the 15th century (p. 22-23 in [30], p. 20-29 in [33]). As one possible indication of this, Roko i Tua is an ancestor in multiple southern *whakapapa* from early migrants Ngāti Māmoe (v.24 p. 33 in [27], p. 15, 23 in [30], p. 175-76 in [32]). As well, the northern Rongo i Amo and Kahukura versions (discussed below) of the Roko i Tua myth are prominent among Ngāti Porou of east coast Te Ika-a-Māui who are cognate with Ngāi Tahu (p. 68-69 in [28]; p. 135-82 in [32]). Assuming these links, the migrants may have situated themselves mythically in southern landscapes by adapting northern traditions over time for new cultural and environmental circumstances (p. 16 in [30], p. 176-77 in [32]). Consistently, southern Ārai Te Uru *waka* traditions lack descendant *whakapapa*, unlike other Ngāi Tahu *waka* narratives (v.26 p. 76 in [27], p. 177 in [32]).

**4. *Kūmara* at Waikouaiti *pā*, Pā a Te Wera**

Separate traditions document a North Otago *kūmara* presence at “Waikouaiti” *pā* (fortified settlement), <20km north of Pūrākaunui, in the forms of *atua*, crop ritual, and stores. The Ngāi Tahu account of Te Rua Pu from Te Taumutu as recorded by John White in the 19th century describes the symbol of a *kūmara* Rongo *atua* kept at Waikouaiti *pā* (p. 185-87 in [24 v.3]). White’s text grounds this *atua* in Murhiku by citing a southern Māori version of his name, “Roko-nui-a-tau”, before translating the full title as “Rongo, the god of the kumara, of great fame through all the year”. The narrative documents a siege of Waikouaiti *pā* by warriors from Te Taumutu. During the siege, Te Rua Pu of the attackers “entered the water which ran round part of the pa” and stole “the symbol” of Roko Nui a Tau away to “the attacking force” (p. 186 in [24 v.3]). After discovering that protective Rongo, “this weapon”, had been stolen, the “priest” of Waikouaiti *pā* Tara i Tū cleared a “small space of the ground” to “resemble a cultivation” and made mounds “similar to the mounds made for the kumara-crop, in honor of Rongo, the god he had lost”. From Te Taumutu, Rongo “felt sorrow” for Tara i Tū and the Waikouaiti people, as “the priest felt sorrow for the god”. Rongo then returned supernaturally to a ceremonial welcome at Waikouaiti (p. 186-87 in [24 v.3]).

White’s reliability in reporting some Māori traditions has been called into question (e.g. p. 20-21, 174 in [31], [79]). Allowing this, there are no reasonable grounds to dispute the narrative of Te Rua Pu at least as collected by White. Credible components include Roko as the southern name for Rongo; (Te) Taumutu and Waikouaiti as Te Waipounamu localities with independent *kūmara* associations (e.g. p. 303 in [16], p. 138 in [33], and sources below), and the site theme of a protective *kūmara atua* who is kept at Waikouaiti *pā*, stolen during a siege, and then returns supernaturally (cf. Kahukura, below). Tara i Tū’s mimetic construction of a ritual garden references a widespread Māori ceremonial practice (p. 116-119 in [14]). This is documented among Ngāi Tahu of the late 19th century Canterbury region where Stack described the *taumatua* or “shrine” enclosure where the *kūmara* crop was consecrated. Stack characterized the *taumatua* or “the god’s garden” as a shrine “a few feet square” that incorporated four mounds “made and planted with kumeras” (p. 24-25 in [81]; see also p. 119 in [14]). In Māori *kūmara* ritual in general, the *atua* of these shrines, especially Rongo, were known as *taumata atua*, who would protect the *mauri* or life force of the crop (p. 202-10 in [14]). In that respect, Tara i Tū’s ritual involving Roko Nui a Tau recalls the active, mythic relationship between installed Rongo/Roko *atua* and *kūmara* cultivations [21].

Some of these themes recur in a 1906 history that references the same “Waikouaiti” *pā* it seems at the Waikouaiti River mouth. At this last location, the *pā* complex known as Huriawa Peninsula (Fig 1, Schedule 103 in [80]) represents the only defended earthworks of archaeological record in the Waikouaiti area with a water border, conforming with the *pā* of White’s Te Rua Pu narrative (p. 186 in [24 v.3] cf. p. 220-25 in [82], p. 62-63, 65, 95 in [83]). James Cowan had gathered “fragments” of the history that “connected” traditional 18th century leader Te Wera and his headland *pā* at Huriawa (Te Pā a Te Wera - the *pā* of Te Wera - also known as Te Pā Katata) from “grey haired Kaumatuas [=elders]” of two North Otago localities [26]). Cowan’s history is focused on the Huriawa siege by Te Wera’s relative Taoka, interpreted by Cowan as occurring “sometime about the year 1770” [26] (p. 50 in [30], Schedule 103 in [80], p. 219-20 in [82]). This chronology places the siege decades before the introduction of cool-climate *Solanum* potato to Murihiku (p. 72-75 in [30]).

One of the traditions that Cowan connected refers to “the cultivations” of Pā a Te Wera below the peninsula. Cowan’s synthesized narrative also references a protecting *atua* of the *pā*, identified in the form of a raised, carved, wooden image of Kahukura [26], the well-documented rainbow deity (p. 513 in [16], p. 414-15 in [65]). Another source confirms that Te Wera kept Kahukura on the Waikouaiti peninsula (p. 556 in [16]). Kahukura was stolen during the siege of Pā a Te Wera by Taoka’s warriors. But like stolen Roko Nui a Tau, Kahukura returned supernaturally after Hatu and other “priests” of the *pā* recited *karakia* (ritual incantations, prayers) [26]. Kahukura is described as the “supreme god of crops”, and one of three deities who presided in the form of raised posts over “the kumara plantation” according to Ngāi Tahu (and other) accounts. Indeed, Kahukura is said to have transferred *kūmara* to Aotearoa by means of a rainbow bridge in connection with, or even instead of, “Rongo-i-amo” (=Rongo i Tua) and the Horouta *waka*. Furthermore, Kahukura is associated with “Rongonuiatau” (=Roko Nui a Tau) as arbiter of life and death, guardian of travellers and *atua* of divination (p. 161 in [23], p. 40 in [24 v.1], p. 102-03 in [24 v.3], p. 68-69, 138-44 in [28], p. 40 in [64], p. 414-15 in [65]) [66]. In short, there are explicit *kūmara* links to Kahukura as a protecting *atua* of Pā a Te Wera, as well as associations with Roko Nui a Tau.

And yet, in spite of Cowan’s references to Kahukura, to “cultivations” below the *pā*, and to surface depressions from Pā a Te Wera that indicate “ancient … food-pits”, one must consider that *kūmara* was not listed by Cowan among the “principal articles of food” stored in preparation for Taoka’s siege. Cowan recorded only “preserved birds”, along with “fern root, and dried fish” [26] among *pā* stores. This inconsistency may reflect variation in Cowan’s sources. In any case, Cowan’s history can be supplemented by Carrington’s reference to Te Wera who took “the precaution,” in anticipation of Taoka’s siege, of having his *pā* “well stocked with food - fern-root, kūmara, and potted bird and fish” (p. 138 in [33]). Carrington’s history is regarded highly for its integrity and general fidelity to his 1930s sources by Ngāi Tahu scholars (e.g. p. 26 in [30], [32], p. 7-19 in [33]).

It is of particular note that Carrington lists *kūmara* alongside the three “principal”, non-domestic food types that Cowan’s source(s) identified as foods stored also at the *pā* [26]. Both lists assume that these foods were available locally so that Pā a Te Wera could be “well stocked” before Taoka’s siege. Since Cowan’s account was published before Carrington compiled his history, *kūmara* becomes all the more conspicuous in Carrington’s list, especially since *kūmara* is not a local food of documentary Murihiku ethnography [16, 30]. It seems unlikely that Carrington, or his Ngāi Tahu source(s), would have embellished this history casually with an anomalous southern food of such cultural prominence. Accordingly, Carrington’s *kūmara* store detail at Huriawa is considered a deliberate addition to the record to preserve a neglected, or alternative, social memory.

**Additional references**

79. Reilly MPJ. John White. Part II: seeking the elusive Mōhio: White and his Maori informants. NZ J History 1990; 24:45–55.

80. Ngai Tahu Claims Settlement Act 1998, New Zealand Legislation. Wellington: New Zealand Parliamentary Counsel Office; 2020. Available from:

http://www.legislation.govt.nz/act/public/1998/0097/latest/DLM429090.html

81. Stack JW. Kaiapohia: The Story of a Siege. Christchurch and Dunedin: Whitcombe & Tombs; 1893 [1990]. Available from: http://www.enzb.auckland.ac.nz/document/?wid=5479&page=0&action=null

82. Brailsford B. The Tattooed Land: The Southern Frontiers of the Pa Maori. Wellington: Reed, 1981.

83. Hamel J. The Archaeology of Otago. Wellington: Department of Conservation, 2001. Available from:

https://www.doc.govt.nz/globalassets/documents/science-and technical/The_Achaeology_of_Otago_Jill_Hamel_WEB.pdf
