# Supplementary material for: Archaeological science meets Māori knowledge to model pre-Columbian sweet potato (Ipomoea batatas) dispersal to Polynesia’s southernmost habitable margins
Source: PLoS One. 2021 Apr 14;16(4):e0247643. doi: 10.1371/journal.pone.0247643 (PMC8046222; doi:10.1371/journal.pone.0247643)
Supplement: S2 Text — (DOCX) [file pone.0247643.s011.docx]

Supplementary 2 Text for:

**Archaeological science meets Māori knowledge to model pre-Columbian sweet potato (*Ipomoea batatas*) dispersal to Polynesia’s southernmost habitable margins**

Ian G. Barber^1*^, Thomas F. G. Higham^2^

^1^ Archaeology Programme, School of Social Sciences, Division of Humanities, University of Otago, Dunedin, New Zealand

^2^ Oxford Radiocarbon Accelerator Unit, Research Laboratory for Archaeology and the History of Art, School of Archaeology, University of Oxford, Oxford, United Kingdom

* Corresponding author: ian.barber@otago.ac.nz (IGB)

**Local marine ΔR and Bayesian modeling with outlier analysis for ^14^C ages from I44/21**

This supplementary text includes information on the determination of a local marine ΔR value and construction of a Bayesian model with outlier detection to analyze the I44/21 ^14^C dataset of our study.

**Local marine ΔR**

As reported in the main article, we applied a local ΔR value of -162±28 for the calibration of mollusc ages in global calibration curve Marine20 [44]. This Δ*R* is the weighted mean of 18 individual marine ^14^C values reported for molluscs of known species and age of death. The mollusc samples of these values were collected between 1855-1955 from marine waters of South Island/Te Waipounamu and the adjacent southern North Island/Te Ika-a-Māui coast (collectively representing central to southern Aoteaora) [84]. Individual sample locations lie within 620km of I44/21, the closest of which is 150km south on the same coast as Pūrākaunui. The 18 Δ*R* values of the mean had been updated for use with Marine20 (S2 Text, Table 1 and [84]; for individual sample details and locations see [85-87]).

We acknowledge that this regional offset value represents a period-specific Δ*R* mean. It is defined here for the relative improvement of I44/21 calibrations only, anticipating further adjustment. On the last, one should allow that temporal change in the southern Pacific marine ^14^C reservoir may have affected Aotearoa coastal waters within the last 700 years [88]. For our dataset, we expect that Bayesian analysis of paired marine and short-life atmospheric samples with outlier detection (discussed below) should control the effects of unrecognized temporal Δ*R* variance in the posterior marine determinations.

**Table 1.** Individual marine ^14^C sample details and values for the weighted mean ΔR of this study (-162±28) with reference to Marine20 [44, 84].

| **Lab ID** | **Locality^a^, km to I44/21** | **Yr collected** | **ΔR^b^** | **σ** | **Mollusc taxon^c^** |
| --- | --- | --- | --- | --- | --- |
| NZ2439 | Pounawea, TW, 150 | 1955 | -176 | 39 | *Leukoma crassicosta* |
| NZ1481 | Kairaki, TW, 300 | 1954 | -109 | 35 | *Paphies subtriangulata* |
| NZ1799 | Paekakariki, sTIM, 531 | 1923 | -194 | 46 | *Dosinia anus* |
| NZ2421 | Makara Beach, sTIM, 543 | 1954 | -181 | 62 | *Haliotis* sp. |
| NZ2433 | Makara Beach, sTIM, 543 | 1954 | -139 | 62 | *Cellana* sp. |
| NZ2431 | Makara Beach, sTIM, 543 | 1954 | -213 | 44 | *Austrovenus stutchburyi* |
| NZ8140a | Turakirae Head, sTIM, 592 | 1855 | -177 | 47 | *Haliotis iris* |
| NZ8140b | Turakirae Head, sTIM, 592 | 1855 | -184 | 41 | *Haliotis iris* |
| NZ8213 | Turakirae Head, sTIM, 592 | 1855 | -132 | 43 | *Thylacodes zelandicus* |
| NZ8214 | Turakirae Head, sTIM, 592 | 1855 | -161 | 35 | *Haustrum haustorium* |
| NZ8215a | Turakirae Head, sTIM, 592 | 1855 | -160 | 36 | *Diloma nigerrimum* |
| NZ8215b | Turakirae Head, sTIM, 592 | 1855 | -176 | 48 | *Diloma nigerrimum* |
| NZ8270 | Turakirae Head, sTIM, 592 | 1855 | -131 | 44 | *Diloma aethiops* |
| NZ8271 | Turakirae Head, sTIM, 592 | 1855 | -141 | 29 | *Lunella smaragda* |
| NZ8272a | Turakirae Head, sTIM, 592 | 1855 | -198 | 45 | *Cellana denticulata* |
| NZ8272b | Turakirae Head, sTIM, 592 | 1855 | -196 | 50 | *Cellana denticulata* |
| NZ1813 | Collingwood R. Mouth, TW, 599 | 1949 | -171 | 46 | *Austrovenus stutchburyi* |
| NZ4698 | Pauatahanui Inlet, sTIM, 620 | 1954 | -152 | 32 | *Alcithoe arabica* |

^a^ TW=Te Waipounamu/South Island; sTIM =southern Te Ika-a-Māui/North Island. For further locality, environment and sample information see [84-87].

^b^ Individual ΔR values updated for Marine20 after [84].

^c^ Binomial nomenclature updated after [89], cf. [84-87].

**Bayesian age model**

The Bayesian model of our study with outlier detection uses the two recent calibration curves SHCal20 and Marine20 with a local historic ΔR as discussed above [43, 44]. This approach follows a chronometric archaeology research method that has proved helpful to resolve shorter archaeological sequences of the later Holocene in particular [67, 90-96].

Our Bayesian model incorporated all ^14^C determinations from the I44/21 S investigation except for the uppermost (L2u) AMS charcoal ages NZA 60804 and NZA 62297 (S2 Table). The last were rejected because of sample proximity to original ground surface below modern aeolian sand sediment. This raised the possibility of intrusion into L2u, suggested independently by the anomalous modern ^14^C age for NZA 62297 (S2 Table). In contrast, the two accepted short-life, single-entity L2u marine samples Wk-44569 and Wk-44570 are *in situ* mollusc valves excavated from an undisturbed, primary position of articulation in the archaeological deposit. Valve articulation in these samples confirmed the stratigraphic integrity of L2u.

Our Bayesian-modeled determinations have been weighted separately for outlier analysis in two OxCal models. A Charcoal model applied only to the 14th century age of unmodeled Wk-37501 from L4 was defined with a prior outlier probability of 1.00 (100%) [45, 91]. As a *Melicytus* sp. fragment of unidentified part, sample Wk-37501 could be from the inner trunk of hardy *M. ramiflorus*, a small lowland forest tree found throughout Aotearoa that may live to “a considerable age” (p. 118 in [97], p. 411 in [98]). This inbuilt age possibility becomes more likely when one compares the three calibrated short-life twig determinations unmodeled from L4 that fall within the 15th century entirely at 95% probability (S2 Table). Setting the prior probability value for Wk-37501 to 100% excludes any posterior influence in the model. A General t-type model was applied to the 21 other ages of our analysis [45]. The General model was defined to detect and downweigh outliers of significance beyond a prior probability of 0.05 (5%). Of these 21 ages, atmospheric NZA 62298 from L4 only was identified as a significant outlier with a posterior probability of 26% (Fig 5). This means that NZA 62298 was ignored in 26% of the model runs that generated posterior distributions.

**Additional references**

84. Stuiver M, Reimer PJ, Reimer RW. 14CHRONO Marine20 Reservoir Database. 2021 [Cited 1 March 2021]. Available from: http://calib.org/marine

85. McFadgen BG. Radiocarbon in modern marine shells compared with the New Zealand standard. N Z J Sci. 1978;21: 311–313.

86. McFadgen BG, Manning MR. Calibrating New Zealand radiocarbon dates of marine shells. Radiocarbon 1990;32: 229–232. doi: 10.1017/S0033822200040194

87. McSaveney MJ, Graham IJ, Begg JG, Beu AG, Hull AG, Kim K, Zondervan A. Late Holocene uplift of beach ridges at Turakirae Head, south Wellington coast, New Zealand. N Z J Geol. Geophys. 2006;49: 337–358. doi: 10.1080/00288306.9515172

88. Petchey F, Schmid MME. Vital evidence: Change in the marine ^14^C reservoir

around New Zealand (Aotearoa) and implications for the timing of Polynesian

settlement. Sci Report 2020;10: 14266. doi: 10.1038/s41598-020-70227-3

89. WoRMS Editorial Board. World Register of Marine Species. 2021. Available from: http://www.marinespecies.org at VLIZ [Cited 1 March 2021]. doi:10.14284/170

90. Bronk Ramsey C. Bayesian analysis of radiocarbon dates. Radiocarbon 2009;51: 337–360. doi: 10.1017/S0033822200033865

91. Dee MW, Bronk Ramsey C. High-precision Bayesian modeling of samples susceptible to inbuilt age. Radiocarbon 2014;56: 83-94. doi: 10.2458/56.16685

92. Bayliss A. Quality in Bayesian chronological models in archaeology. World Archaeol. 2015;47: 677–700. doi: 10.1080/00438243.2015.1067640

93. Burley D, Edinborough K, Weisler M, Zhao J-x. Bayesian modeling and chronological precision for Polynesian settlement of Tonga. PLoS ONE 2015;10(3): e0120795. doi: 10.1371/journal.pone.0120795

94. Marsh E, Kidd R, Ogburn D, Durán V. Dating the expansion of the Inca Empire: Bayesian models from Ecuador and Argentina. Radiocarbon 2017; 59: 117-140. doi: 10.1017/RDC.2016.118

95. Hamilton W, Krus A. The myths and realities of Bayesian chronological modeling revealed. Am Antiq. 2018;83: 187–203. doi: 10.1017/aaq.2017.57

96. Manning SW, Hart JP. Radiocarbon, Bayesian chronological modeling and early European metal circulation in the sixteenth-century AD Mohawk River Valley, USA. PLoS ONE 2019;14(12): e0226334. doi: 10.1371/journal.pone.0226334

97. Salmon JT. The Native Trees of New Zealand. Rev. ed. Wellington: Heinemann Reed, 1986.

98. Dawson J, Lucas R. New Zealand’s Native Trees. Rev. ed. Nelson: Potton & Burton, 2019.

**S2 Text Appendix. Bayesian Model CQL code.**

Plot()

{

Curve("SHCal20","SHCal20.14c");

Curve("Marine20","Marine20.14c");

Delta_R("LocalMarine",-162,28);

Outlier_Model("General",T(5),U(0,4),"t");

Outlier_Model("Charcoal",Exp(1,-10,0),U(0,3),"t");

Sequence()

{

Boundary("Start L4");

Phase("L4")

{

Curve("=SHCal20");

R_Date("NZA 60803 ", 473, 19)

{

Outlier("General", 0.05);

};

R_Date("NZA 62298 ", 437, 18)

{

Outlier("General", 0.05);

};

R_Date("Wk-37501 ", 655, 20)

{

Outlier("Charcoal", 1.00);

};

R_Date("Wk-37502 ", 542, 21)

{

Outlier("General", 0.05);

};

R_Date("Wk-37503 ", 521, 20)

{

Outlier("General", 0.05);

};

Curve("=Marine20");

Delta_R("=LocalMarine", );

R_Date("Wk-44574 ", 886, 22)

{

Outlier("General", 0.05);

};

R_Date("Wk-14100", 930, 35)

{

Outlier("General", 0.05);

};

};

Boundary("Transition L4/P3");

Phase("P3")

{

Curve("=SHCal20");

R_Date("Wk-38601", 485, 20)

{

Outlier("General", 0.05);

};

Curve("=Marine20");

Delta_R("=LocalMarine", );

R_Date("Wk-37521 ", 927, 21)

{

Outlier("General", 0.05);

};

R_Date("Wk-37522 ", 907, 20)

{

Outlier("General", 0.05);

};

R_Date("Wk-44573 ", 879, 21)

{

Outlier("General", 0.05);

};

R_Date("Wk-12596", 958, 35)

{

Outlier("General", 0.05);

};

R_Date("Wk-12597", 897, 34)

{

Outlier("General", 0.05);

};

};

Phase("P3cap")

{

R_Date("Wk-37520 ", 889, 20)

{

Outlier("General", 0.05);

};

};

Boundary("Transition 2/3");

Phase("L2")

{

Curve("=SHCal20");

R_Date("NZA 62299 ", 435, 17)

{

Outlier("General", 0.05);

};

R_Date("Wk-37504 ", 492, 22)

{

Outlier("General", 0.05);

};

R_Date("Wk-37505 ", 488, 22)

{

Outlier("General", 0.05);

};

Curve("=Marine20");

Delta_R("=LocalMarine", );

R_Date("Wk-44571 ", 862, 20)

{

Outlier("General", 0.05);

};

R_Date("Wk-44572 ", 896, 20)

{

Outlier("General", 0.05);

};

R_Date("Wk-14099", 868, 35)

{

Outlier("General", 0.05);

};

};

Boundary("Transition L2/L2u");

Phase("L2u")

{

R_Date("Wk-44569 ", 911, 20)

{

Outlier("General", 0.05);

};

R_Date("Wk-44570 ", 873, 22)

{

Outlier("General", 0.05);

};

};

Boundary("End Occupation");

};

Difference("Diff start to end", "End Occupation", "Start L4");

};
